# Dual-colour imaging of RNAs using quencher- and fluorophore-binding aptamers

**DOI:** 10.1093/nar/gkv718

**Published:** 2015-07-14

**Authors:** Ankita Arora, Murat Sunbul, Andres Jäschke

**Affiliations:** Institute of Pharmacy and Molecular Biotechnology, Heidelberg University, Im Neuenheimer Feld 364, Heidelberg 69120, Germany

## Abstract

In order to gain deeper insight into the functions and dynamics of RNA in cells, the development of methods for imaging multiple RNAs simultaneously is of paramount importance. Here, we describe a modular approach to image RNA in living cells using an RNA aptamer that binds to dinitroaniline, an efficient general contact quencher. Dinitroaniline quenches the fluorescence of different fluorophores when directly conjugated to them via ethylene glycol linkers by forming a non-fluorescent intramolecular complex. Since the binding of the RNA aptamer to the quencher destroys the fluorophore-quencher complex, fluorescence increases dramatically upon binding. Using this principle, a series of fluorophores were turned into fluorescent turn-on probes by conjugating them to dinitroaniline. These probes ranged from fluorescein-dinitroaniline (green) to TexasRed-dinitroaniline (red) spanning across the visible spectrum. The dinitroaniline-binding aptamer (DNB) was generated by *in vitro* selection, and was found to bind all probes, leading to fluorescence increase *in vitro* and in living cells. When expressed in *E. coli*, the DNB aptamer could be labelled and visualized with different-coloured fluorophores and therefore it can be used as a genetically encoded tag to image target RNAs. Furthermore, combining contact-quenched fluorogenic probes with orthogonal DNB (the quencher-binding RNA aptamer) and SRB-2 aptamers (a fluorophore-binding RNA aptamer) allowed dual-colour imaging of two different fluorescence-enhancing RNA tags in living cells, opening new avenues for studying RNA co-localization and trafficking.

## INTRODUCTION

The use of intrinsically fluorescent, genetically encoded tags has unveiled intriguing details about the dynamics and localization of many proteins. However, the absence of an effective parallel method to image RNA *in vivo* has impeded the study of RNA dynamics and subcellular localization.

One of the conventional methods for imaging intracellular RNA is to utilize molecular beacons, stem–loop shaped oligo­nucleotides with a fluorophore and a quencher held in close proximity to each other. When the molecular beacons bind to the complementary target RNA, the hairpin configuration is altered to a linear shape which separates the fluorophore and the quencher from each other resulting in fluorescence restoration (1,2). However, problems in delivery, stability and sequestration of probes have limited their use *in vivo* (3). Another method practiced extensively to image RNA utilizes GFP-tagged RNA binding proteins (RBP) that recognize specific RNA motifs (MS2 (4,5), λ_N_ (6) and PUMILIO1 (7)). The RNA of interest (ROI) is tagged with multiple copies of an RNA motif with which the GFP-fused RBP can associate, rendering the resulting RNA–protein complex fluorescent. However, the large size of the GFP tag may interfere with the migration or alter the function of the RNA (3).

Thus, there is a need to develop small-molecule based RNA imaging methods as they offer better cellular permeability, low toxicity, structural flexibility and high multiplexing potential. Previous attempts using systematic evolution of ligands by exponential enrichment (SELEX) (8) have been made to identify small RNA sequences (aptamers) that bind to fluorogenic dyes (with low or no intrinsic fluorescence) which light up upon binding (9–15). Additionally, aptamers that bind to various fluorophores such as fluorescein (16), sulforhodamine B (16), and tetramethyl­rhodamine (17), have been developed. They could, however, not be used for *in vivo* applications, primarily due to high background fluorescence. Recently, an aptamer (Spinach) that binds to 3,5-difluoro-4-hydroxybenzylidine imidazoli­none (DFHBI), a derivative of the GFP chromophore, has been developed and used for *in vivo* RNA imaging, which was the first successful attempt to image RNA with small molecules in live cells (18–20). Another recent approach utilizes IMAGE (Intracellular MultiAptamer GEnetic) tags, containing tandem repeats of tobramycin binding aptamer, and Cy3-tobramycin/Cy5-tobramycin probe pair. Using IMAGE, RNA transcription was followed in live yeast cells in the presence of Cy3-tobramycin/Cy5-tobramycin probes by detecting FRET signal between Cy3 and Cy5 fluorophores (21). Lately, we developed a new strategy combining contact-quenched fluorescent turn-on probes and a fluorophore-binding aptamer to image RNA in living cells. We synthesized various contact-quenchers conjugated to the sulforhodamine B fluorophore to image RNAs of interest fused to the sulforhodamine B binding aptamer (SRB-2). The fluorescence of the probes was found to increase drastically upon binding to SRB-2, and dinitroaniline was found to be the best contact-quencher for sulforhodamine B (22). Contact quenching is a type of static quenching where the fluorophore and the quencher interact with each other to form a non-fluorescent intramolecular dimer with its own distinct absorption spectrum. Therefore, the quenching efficiency is independent of the absorption and emission spectra of the quencher and the fluorophore, unlike FRET quenching (23). The advantage of contact quenching over conventional FRET-(24) or PeT-(25) based systems is that it can be completely abolished by disrupting the complex between fluorophore and quencher upon binding of the aptamer to the fluorophore, while FRET or PeT quenching could not be fully suppressed. Thus, contact-quenched probes may result in more effective fluorescence restoration than FRET- or PeT-quenched probes. However, all the above mentioned techniques are restricted by the limited number of fluorophores that can be used in the experimental setup as an aptamer needs to be developed against each fluorophore to be used.

**Figure 1.**
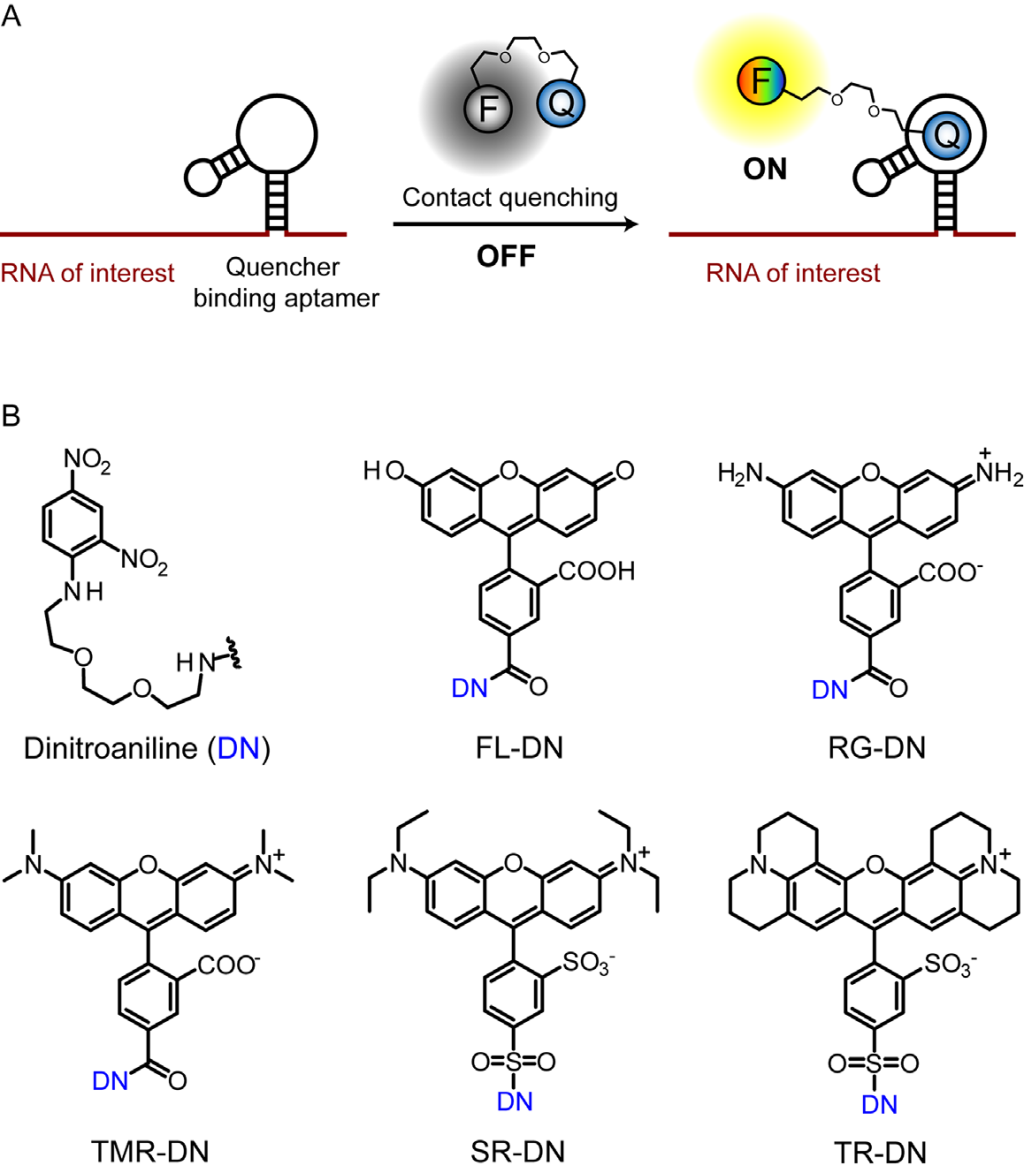
Scheme for imaging RNA by using contact-quenched fluorogenic probes. (**A**) The contact-quenched fluorophore-dinitroaniline conjugates (OFF) light up upon binding to the quencher binding aptamer (ON). RNA of interest can be fused to the quencher binding aptamer and imaged in the presence of the fluorophore-quencher conjugate. F denotes any fluorophore and Q denotes a contact quencher. (**B**) Structures of the contact quencher and fluorogenic probes used in this study. Dinitroaniline (DN) is the contact-quencher used in this work. FL-DN: fluorescein-dinitroaniline, RG-DN: rhodamine green dinitro­aniline, TMR-DN: tetramethylrhodamine-dinitro­aniline, SR-DN: sulforhodamine-dinitroaniline, TR-DN: TexasRed-dinitroaniline.

Therefore, shifting from a fluorophore-binding aptamer approach to a quencher-binding aptamer system would help to overcome the limitations of previous approaches as we are no longer restricted with the limited number of available fluorophore-binding RNAs (Figure 1A). In addition, the independence of the RNA–quencher interaction from the structure of the fluorophore opens the door for multiplexing and imaging multiple RNAs simultaneously when combined with the other RNA imaging methods. Here, we describe the development of a robust, small-molecule based method for RNA imaging *in vivo* that combines the advantages of contact quenching and fluorescence restoration by a quencher-binding RNA. We chose dinitroaniline as the contact quencher, since it was previously identified as a general and efficient quencher for a variety of fluorophores with a high fluorescence enhancement upon disruption of the fluorophore-quencher interaction (22,26).

We synthesized various fluorogenic probes spanning the visible spectrum by coupling dinitroaniline with different fluorophores via triethylene glycol linkers, thereby forming non-fluorescent complexes (OFF-state). These probes were fluorescein-dinitroaniline (FL-DN), rhodamine green-dinitro­aniline (RG-DN), tetramethylrhodamine-dinitro­aniline (TMR-DN), sulforhodamine-dinitroaniline (SR-DN) and TexasRed-dinitroaniline (TR-DN) (Figure 1B). We hypothesized that in the presence of a quencher-binding aptamer, the quencher would preferentially interact with the aptamer, thereby losing contact with the fluorophore and giving rise to fluorescence enhancement (ON-state) (Figure 1A). Here, we report the generation of an aptamer against the dinitroaniline quencher, and its application as a fluorescence-enhancing genetically encoded tag for RNA imaging in live *Escherichia coli* in combination with the new probes.

## MATERIALS AND METHODS

### General materials and methods

Unless otherwise stated, chemicals were purchased from Sigma–Aldrich, Thermo Scientific or Invitrogen and used without further purification. Reactions were typically performed in anhydrous solvents and under normal atmosphere. The reaction progress was monitored by TLC, visualized under UV-light or by staining with blue-shift reagent. Reverse phase HPLC purifications were performed on a Lobar^®^310-25 LiChroprep^®^ RP-18 (40–63 μm) column (Merck) and compounds were eluted with a mixture of acetonitrile and water containing 0.1% trifluoroacetic acid. MALDI-TOF mass spectrometry (Bruker Biflex III mass spectrometer, 2,5-dihydroxybenzoic acid as a matrix) was used to monitor the progress and success of the reactions. High resolution mass spectra were recorded on a Bruker microTOFQ-II ESI mass spectrometer. RNA concentrations were determined by a Qubit^®^ 2.0 fluorometer (Life Technologies). Fluorescence measurements were performed with a JASCO FP-6500 spectrofluorometer. Absorbance spectra were recorded on a Cary 50 UV-Vis spectrophotometer (Varian). Agarose gels were stained with ethidium bromide and visualized by UV illumination using an AlphaImager™ 2200.

### *In vitro* selection (first SELEX, rounds 1–8)

*In vitro* transcriptions were performed as follows: 0.5 μM of dsDNA template was added in transcription buffer (40 mM Tris pH 8.1, 1 mM spermidine, 22 mM MgCl_2_, 0.01% Triton-X-100) along with 10 mM DTT, 0.01 mg/ml BSA, 4 mM of ATP, CTP, UTP and GTP. The transcription reaction was doped with 50–100 μCi of radioactive CTP (^32^P α-CTP, 10 μCi/μl, Hartmann Analytics, Germany). 1 μM of T7 RNA polymerase (lab prepared stock) was added and the transcription reaction was incubated at 37°C for 4 h. The transcription reaction was purified on a 10% denaturing polyacrylamide gel, eluted in 0.3 M Na-acetate (pH 5.5) overnight and iso-propanol precipitated. The pellets were washed with 75% ethanol and dissolved in Millipore water. The RNA was folded prior to the binding step by incubating the RNA at 75°C for 2 min, followed by controlled cooling to 25°C over 15 min. Then, one-fifth volume of 6X selection buffer (120 mM Hepes, 30 mM MgCl_2_ and 750 mM KCl, pH 7.4) was added and the aptamer was incubated at 25°C for additional 15 min. To remove RNAs that bound to the affinity resin, the RNA library was first incubated with mock resin for 30 min at 25°C. The unbound RNA obtained after mock treatment was incubated with dinitroaniline-(PEG)_3_-amine-functionalized resin for 30 min at 25°C. The resin was washed with 12 volumes of 1X selection buffer and bound RNA was eluted with 5 mM EDTA by heat denaturation. The bound RNA was ethanol precipitated and dissolved in MilliQ water. For the reverse transcription (RT) reaction, 5 μM primer B and 0.5 mM dNTPs were added to the dissolved RNA and heated at 65°C for 5 min. After cooling down on ice, 5 mM DTT, 1X first-strand buffer (Invitrogen, 50 mM Tris, pH 8.3, 75 mM KCl, 3 mM MgCl_2_) and 10 U/μl of RT enzyme (SuperScript III, Invitrogen) were added and the reaction was incubated at 53°C for 1 h. For PCR amplification of the selected species, the following reagents were added directly to the reverse transcription reaction: 1X PCR buffer (Rapidozyme; 67 mM Tris, pH 8.8, 16 mM (NH_4_)_2_SO_4_, 0.01% Tween 20), 5.25 mM MgCl_2_, 5 μM forward primer and 3.75 μM reverse primer, 0.375 mM of each dNTP and 0.1 U/μl Taq polymerase (Rapidozyme). DNA was amplified under the following conditions: 94°C/1 min, 52°C/1 min, 72°C/1 min with nine additional cycles from step 2 and a final amplification at 72°C/20 min. Prior to the start of the next round, the RT-PCR product was purified via a Qiagen PCR purification kit.

### First SELEX (rounds 9–15) and second SELEX

In order to increase the stringency of the selection for evolving high-affinity binders, biotinylated dinitroaniline was synthesized (DN-SS-Biotin, see Supplementary Information) to allow for a sequential decrease in the amount of ligand in each round. The concentration of the biotinylated ligand used in round 9 was 100 μM and was decreased subsequently in next rounds. Streptavidin, immobilized on Agarose CL-4B (Fluka) was used as the solid support for affinity binding. The SELEX protocol was the same as described above except that the elution was performed specifically with 20 mM DTT which cleaves the dithiol group present in the biotinylated ligand (DN-SS-Biotin), releasing the resin-bound RNA while biotin remains bound to the streptavidin resin.

### *In vitro* transcription protocol

Template DNA for wt-DNB or DNB aptamer (0.5 μM) was added into a transcription mixture containing NTPs (4 mM each), DTT (10 mM), spermidine (1 mM), Tris–HCl (40 mM, pH 8.1), MgCl_2_ (22 mM), Triton-X-100 (0.01%), BSA (40 μg/ml), pyrophosphatase (1 U/ml) and T7 RNA polymerase (1 μM, lab prepared stock). Transcription was carried out for 4 h at 37°C, and then the reaction mixture was treated with DNaseI (50 U/ml) for 30 min at 37°C. RNA was purified by electrophoresis on a 10% denaturing polyacrylamide gel and excised from the gel. The excised gel was incubated in sodium acetate buffer (0.3 M, pH 5.5) overnight. Eluted RNA was ethanol-precipitated and dissolved in MilliQ water. RNA samples were stored at −20°C.

### Determination of dissociation constants

Dissociation constants (*K*_D_) for the RNA–probe complexes were determined by measuring the increase in fluorescence as a function of increasing RNA concentration in the presence of a fixed amount of the probe (100 nM) at 25°C. Just before the measurements, RNA was folded in the selection buffer as mentioned before. The excitation (Ex) and emission (Em) wavelength used for various probes were as follows: FL-DN (Ex: 498 nm, Em: 528 nm), RG-DN (Ex: 507 nm, Em: 534 nm), TMR-DN (Ex: 555 nm, Em: 582 nm), SR-DN (Ex: 572 nm, Em: 591 nm) and TR-DN (Ex: 598 nm, Em: 619 nm). A bandwidth of ±5 nm for both excitation and emission filters was used in all measurements.

The dissociation constant of dinitroaniline-amine was determined in a competition experiment where the DNB aptamer (100 nM) was first saturated with SR-DN (1 μM) and the decrease in fluorescence was measured as a function of increasing concentration of dinitroaniline-amine. The IC_50_ was determined by fitting the curve to the Hill equation and *K*_D_ was calculated as described in the literature (27).

### Determination of quantum yields

Sulforhodamine 101 (28) and fluorescein (29,30), having quantum yields of 1.00 in ethanol and 0.925 in 0.1 M sodium-borate buffer (pH 9.1), respectively, were used as the standards for measuring quantum yields. The integral of the fluorescence emission spectrum and the absorbance of the probes at the excitation wavelength were measured and plotted in a graph by using four different concentrations of the probes. It is important to ensure that the absorbance values at the different concentrations measured are below 0.05 to avoid inner filter effects during fluorescence measurements. The slope of the linear fit was compared with that of the reference compound, and the quantum yields were calculated by using the following equation:

\begin{equation*} QY = QY_{\rm R} \left( {\frac{m}{{m_{\rm R} }}} \right)\left( {\frac{{n^2 }}{{n_{\rm R}^2 }}} \right) \end{equation*}

where *m* is the slope of the line obtained from the plot of the area under the fluorescence emission spectrum versus absorbance, *n* is the refractive index of the solvent and the subscript R refers to the reference fluorophore of known quantum yield.

### Live cell imaging in *E. coli*

BL21-DE3 star *E. coli* cells (Invitrogen) were transformed with 50 ng of plasmid DNA expressing the tRNA scaffold (31) or the dinitroaniline aptamer sequence (pET28-DNB). Single colonies were picked and incubated overnight in LB medium containing kanamycin (30 μg/ml) at 37°C with shaking at 150 rpm. At OD_600_ = 0.4, IPTG (1 mM) was added and cells were shaken for 3–4 h at 37°C. Then, 0.2 ml of the culture was removed, pelleted, washed and resuspended in 1 ml of live-cell imaging solution (Invitrogen) supplied additionally with 5 mM MgSO_4_ and 20 mM glucose. 400 μl of this suspension was transferred in to a poly-d-lysine-coated 4-well glass chamber and incubated at 37°C for 30 min. The cell suspension was removed and the wells were washed twice gently with the live-cell imaging solution. The cells were incubated with 1 μM of various probes in live-cell imaging solution at 37°C for 5 min. An inverted Nikon eclipse Ti microscope equipped with 100X oil objective was used for imaging and the image was background corrected using ImageJ software.

## RESULTS

### *In vitro* selection of the quencher-binding aptamer

**Figure 2.**
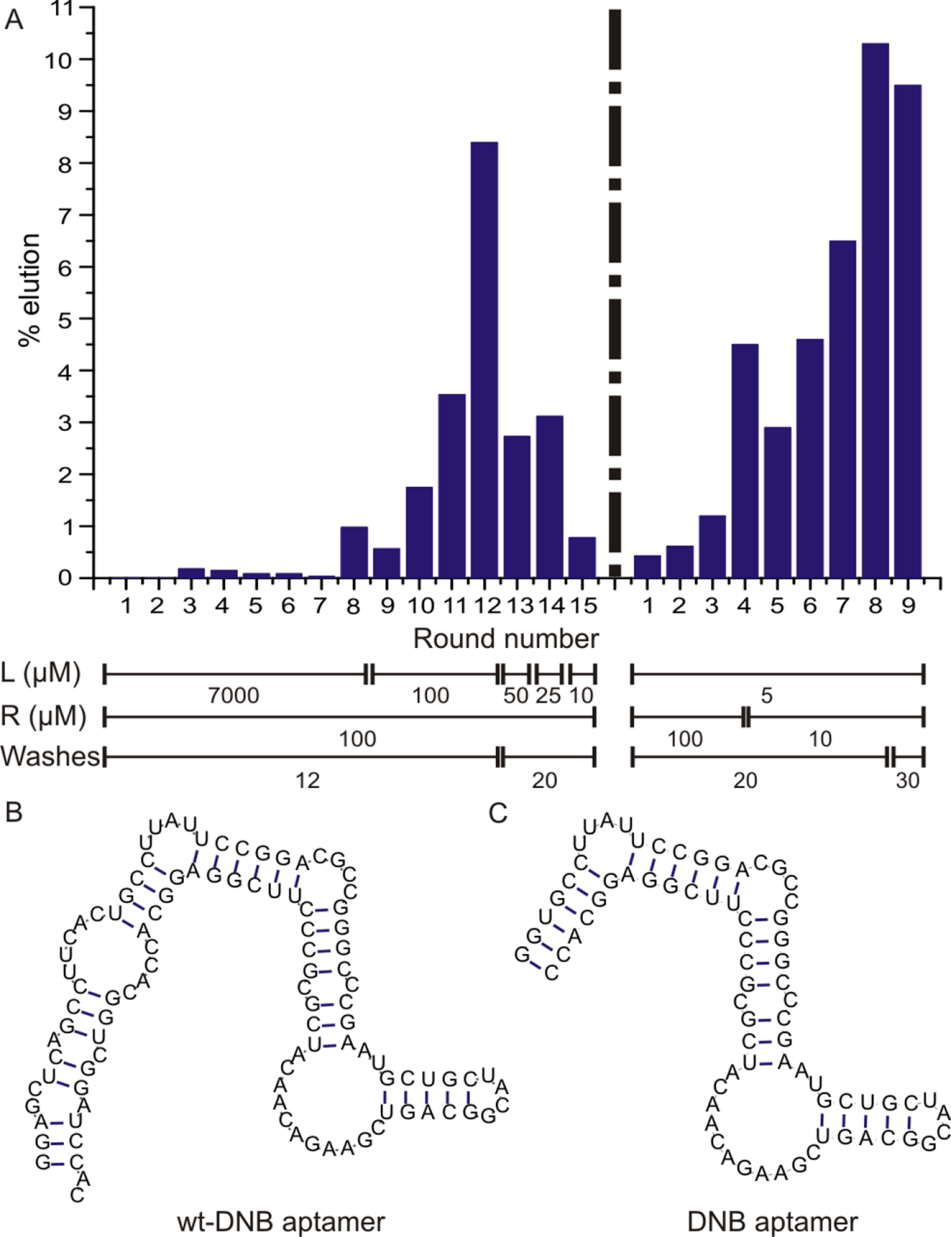
(**A**) Progress of the *in vitro* selection. Selection progress was monitored by calculating% of RNA eluted from dinitroaniline-immobilized resin. L denotes the ligand concentration (μM), R denotes the RNA concentration (μM) and W denotes the number of column washes for each round of SELEX. (**B**) Mfold-predicted secondary structure of wt-DNB aptamer. (**C**) Mfold-predicted secondary structure of DNB aptamer.

*In vitro* selection (SELEX) was performed to evolve RNA aptamers that bind to dinitroaniline with a library containing ∼2 × 10^15^ DNA molecules (Supplementary Figure S1A). We prepared a partially structured DNA library containing two fixed primer binding sites flanking a 64-nucleotide region that consists of two 26-base random stretches separated by a 12-base constant region which forms a stable stem–loop (Supplementary Figure S1B) (32). The DNA pool was transcribed into RNA using T7 RNA polymerase, and the purified transcripts were first incubated with the mock-resin to remove resin-binding aptamers. The unbound RNA was then incubated with the resin functionalized with dinitroaniline for 30 min and the resin-bound RNA species were eluted. These RNAs were then reverse transcribed and PCR-amplified, and the enriched pool obtained served as input for the next round of selection. After 15 rounds of SELEX to evolve dinitroaniline-binding RNA aptamers, the pool was found to weakly enhance the fluorescence of SR-DN. We sequenced about ∼50 individual members from round 15 RNA pool and found that the library was still very diverse and did not converge to different families (Supplementary Table S1). To identify individual fluorescence-enhancing RNA aptamers, transcribed sequences were screened for an increase in fluorescence upon binding to SR-DN. We hypothesized that the binding of the aptamer to the quencher would destroy the non-fluorescent fluorophore-quencher intramolecular dimer complex, resulting in a fluorescence increase. The aptamer with the highest fluorescence enhancement (10-fold, clone 5) discovered from round 15 was further characterized and its affinity to the five fluorogenic probes was found to range from 8 to 53 μM with modest fluorescence enhancement (4–12-fold, Supplementary Figure S2). To obtain efficient labelling in a cellular environment, it is of utmost importance to obtain aptamers with high affinity and very high fluorescence turn-on ratio. Therefore, we decided to prepare a new library and increase the stringency of the selection protocol. The new library was generated by combining a randomly mutagenized enriched pool of round 15 and randomly mutagenized clone 5 aptamer from round 15 in a ratio of 7:3 (for details see Supplementary Information). This way, the sequence diversity was increased and a second SELEX was carried out with increasing selection pressure by decreasing the amount of RNA to be incubated with the substrate and by increasing the number of column washes. After nine rounds of selection, an increase in quencher-bound RNA from 0.43% to 9.5% was observed (Figure 2A) and the DNA pool of round 9 was further analysed.

### Sequence analysis and truncation studies

Out of the 96 colonies that were sequenced, only few sequences were found more than once, indicating that the pool was still diverse (Supplementary Table S2). Again, transcribed sequences were screened against SR-DN to identify the best sequence giving the highest fluorescence enhancement. The best clone identified showed 56-fold fluorescence enhancement when bound to SR-DN with a *K*_D_ of 1.4 μM and was named as wt-DNB (wild-type dinitroaniline-binding aptamer, Figure 2B).

Truncation experiments revealed a 75-nucleotide minimal domain (named DNB), obtained by deleting the primer binding regions from both ends (Figure 2C). Further deletion of the nucleotides from either 5′- or 3′-ends of DNB interfered with the binding of dinitroaniline and significantly decreased the fluorescence enhancement factors. Both wt-DNB and DNB aptamers were found to have similar fluorescence enhancement factors, however DNB had a two-fold higher affinity to SR-DN than the wild type sequence (Supplementary Figure S3). Hence, we focused on DNB for further analysis.

### Characterization of the DNB aptamer

**Figure 3.**
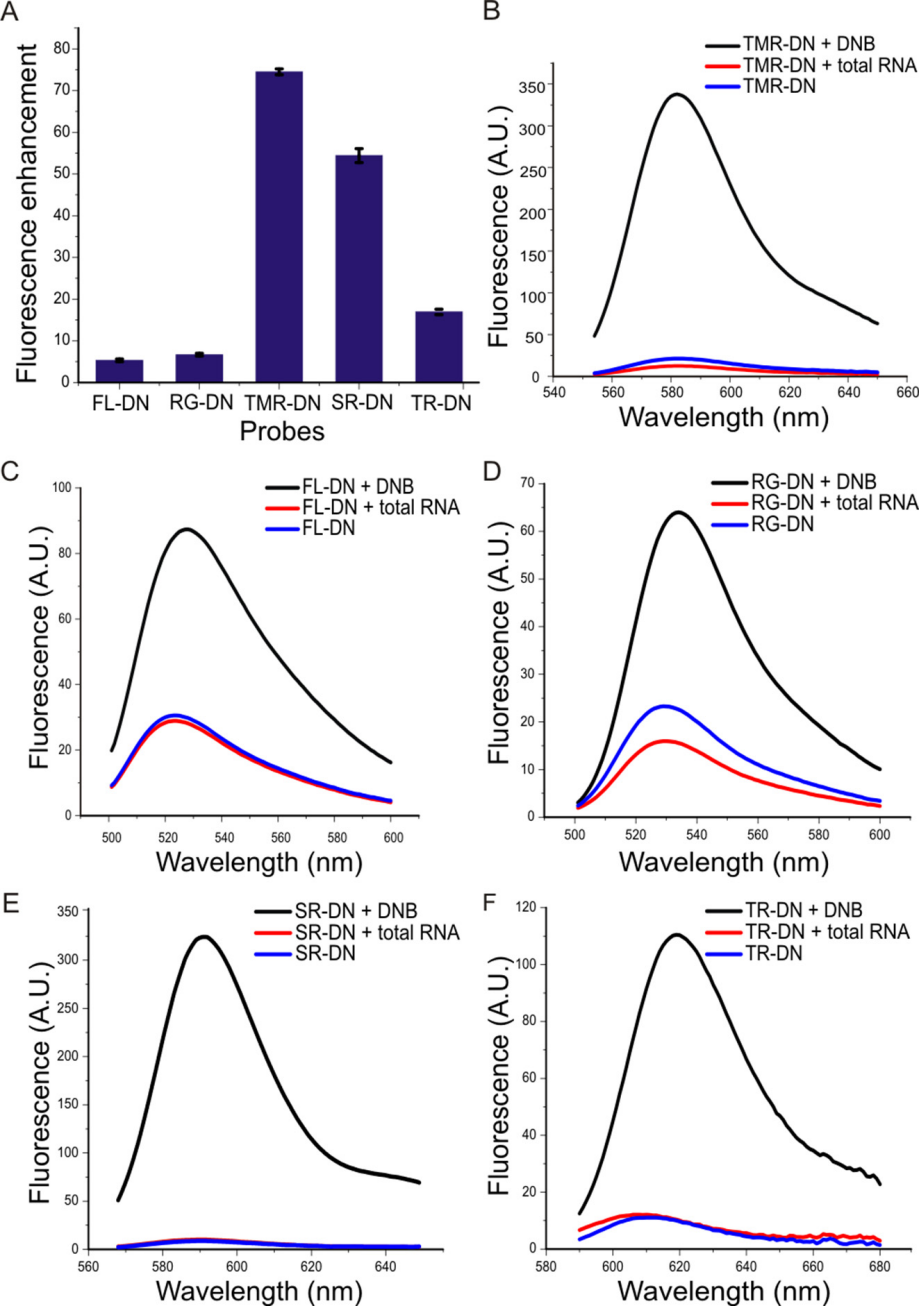
Confirmation of DNB aptamer's ability to restore the fluorescence of various fluorogenic probes. (**A**) Fluorescence enhancement factors (*F*_aptamer:probe_/*F*_total RNA:probe_) obtained upon binding of the DNB aptamer (10 μM) to various probes (1 μM). Fluorescence emission spectra of various probes (1 μM) in the presence of DNB aptamer (10 μM, black), alone (blue) and in the presence of *E. coli* total RNA (red). (**B**) TMR-DN. (**C**) FL-DN. (**D**) RG-DN. (**E**) SR-DN. and (**F**) TR-DN.

The fluorescence enhancement of the various contact-quenched probes (FL-DN, RG-DN, TMR-DN, SR-DN and TR-DN) was evaluated in a binding assay. The fluorescence value of the respective fluorophore-dinitroaniline probe (1 μM) in the presence of excess DNB (10 μM) was measured and divided by the fluorescence of the probe in *E. coli* total RNA. The fluorogenic probes FL-DN, RG-DN, TMR-DN, SR-DN and TR-DN showed 5-, 6-, 73-, 56- and 15-fold fluorescence enhancement upon binding to the aptamer, respectively (Figure 3).

**Figure 4.**
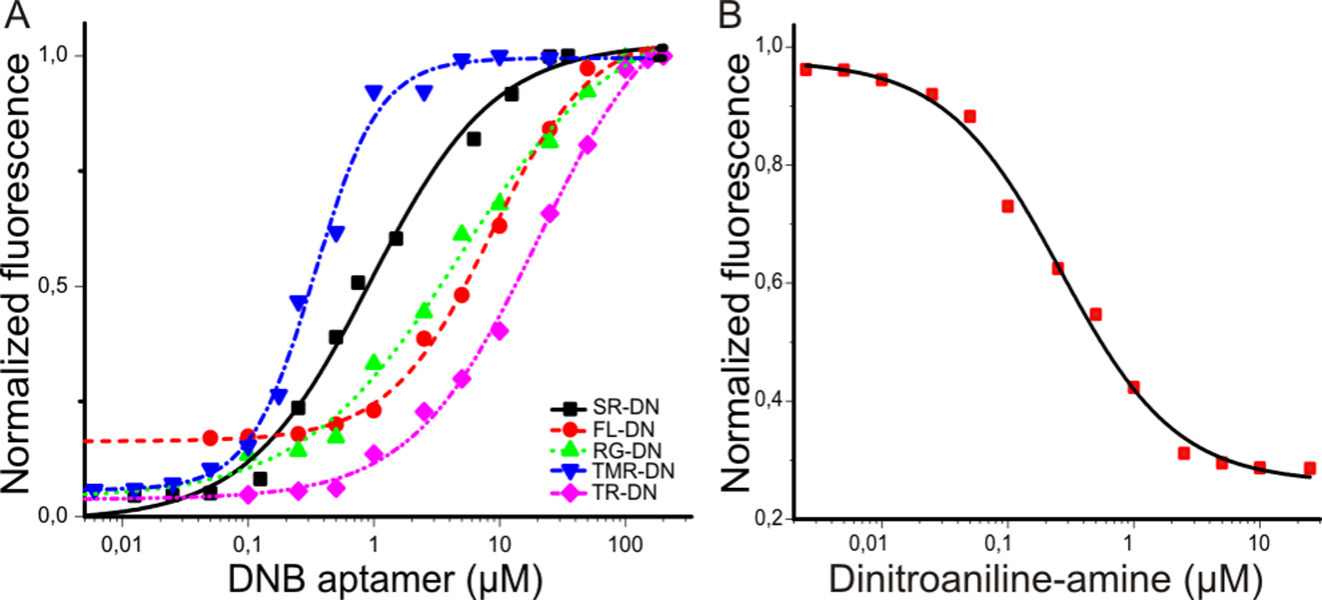
Determination of the binding affinity between various probes and DNB. (**A**) Dissociation constants (*K*_D_) between DNB and various probes. The *K*_D_ values were determined by titrating increasing amounts of aptamer with a fixed concentration of probe (100 nM) and measuring the fluorescence increase upon binding. The *K*_D_ values were found to be 8.55 ± 0.53 μM for FL-DN-DNB (red), 4.48 ± 0.60 μM for RG-DN-DNB (green), 0.35 ± 0.05 μM for TMR-DN-DNB (blue), 0.80 ± 0.1 μM for SR-DN-DNB (black) and 18.0 ± 1.8 μM for TR-DN-DNB (pink). (**B**) The dissociation constant between dinitroaniline-amine (ligand used for SELEX) and DNB was calculated in a competition based assay where the DNB aptamer (100 nM) was saturated with SR-DN (1 μM) and the fluorescence decrease was measured upon addition of dinitroaniline-amine. The *K*_D_ was determined from the IC_50_ obtained by the competition assay. *K*_D_ was calculated to be 100 ± 14 nM.

We also measured the dissociation constants (*K*_D_) between the probes and DNB by titrating a solution of the probes (100 nM) with increasing amounts of DNB (Table 1, Figure 4A). The measured *K*_D_ values varied between 350 nM to 18 μM. These large differences are presumably due to the different strengths of the fluorophore-quencher interactions, as this interaction needs to be destroyed in order for the probe to bind to the DNB aptamer. In addition, we also determined the *K*_D_ between DNB and dinitroaniline-amine (the ligand used for SELEX) in a competition experiment as 100 ± 14 nM (Figure 4B). The affinity of DNB for dinitroaniline-amine is higher than for the probes as there is no intramolecular fluorophore–quencher complex that needs to be broken apart for the binding of the aptamer.

**Table 1.** Spectroscopic properties of free and DNB aptamer-bound probes measured in 1X selection buffer at 25°C

Probe	Ex (nm)	Em (nm)	Extinction coefficient (M^−1^cm^−1^)	*K*_D_ (μM)	Quantum yield	Brightness^a^
FL-DN/DNB	498	528	51 750	8.55 ± 0.53	ND^b^	ND
FL-DN	496	523	63 806		0.051	15
RG-DN/DNB	507	534	37 350	4.48 ± 0.60	0.32	55
RG-DN	502	529	42 849		0.074	15
TMR-DN/DNB	555	582	47 150	0.35 ± 0.05	0.90	195
TMR-DN	554	582	57 206		0.080	21
SR-DN/DNB	572	591	50 250	0.80 ± 0.10	0.98	226
SR-DN	568	589	46 314		0.027	6
TR-DN/DNB	598	619	52 366	18.0 ± 1.8	ND	ND
TR-DN	591	611	45 600		0.086	18
GFP	395	508	27 600	-	0.79	100

^a^Brightness (extinction coefficient × quantum yield) is reported relative to GFP.

^b^ND stands for not determined.

The best probes for the DNB aptamer were found to be TMR-DN and SR-DN, both of which have dissociation constants in the nanomolar range (350 and 800 nM, respectively) and very high fluorescence turn-on ratios (73- and 56-fold, respectively). In addition, we investigated the fluorescence quantum yields of the free probes as well as the DNB-bound probes (Table 1). As expected, dinitroaniline behaved as a general and very efficient contact quencher for the variety of fluoro­phores, and quantum yields of the fluorophores decreased drastically when they were conjugated to dinitroaniline. However, when the probes were bound to the DNB aptamer, the quantum yields of the probes increased owing to the disruption of the fluorophore-quencher intramolecular complex.

Moreover, DNB aptamer was folded in the presence of different concentrations of magnesium ions and the fluorescence emission measured upon addition of TMR-DN. Only the correctly folded fraction of the DNB aptamer would bind to the probe, resulting in a fluorescence increase. The highest fluorescence signal (100%) was observed at 1 mM and higher Mg^2+^ concentrations, and the fluorescence intensity decreased to 80% and 60% in the presence of 0.5 and 0.25 mM magnesium, respectively (Supplementary Figure S4A).

To determine the melting temperature of the DNB aptamer, DNB aptamer and TMR-DN probe were mixed and the fluorescence intensity measured at different temperatures. The melting temperature was determined to be 43°C, indicating that ∼80% of the aptamer is correctly folded when imaged at 37°C (Supplementary Figure S4B).

### RNA imaging in live bacterial cells

**Figure 5.**
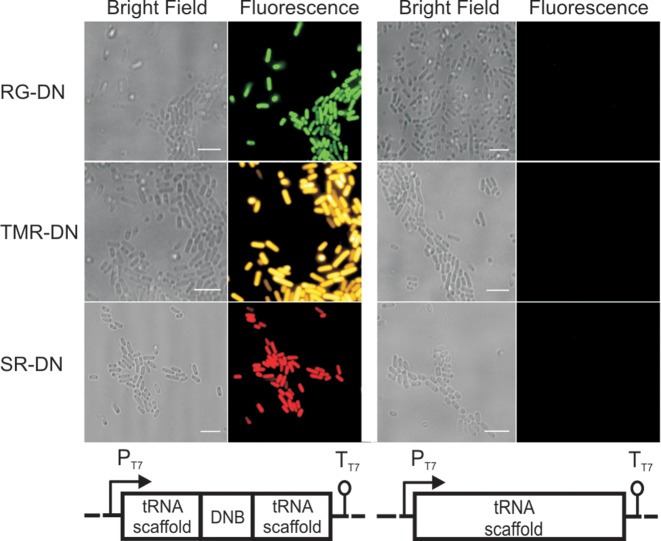
Imaging DNB aptamer in live *E. coli* with various fluorophore-dinitroaniline conjugates. Bacteria were transformed with either pET28-DNB or pET28-tRNA plasmid. Transcription was induced with IPTG. Bacteria were incubated with 1 μM of RG-DN (green), TMR-DN (yellow) and SR-DN (red) for 5 min and imaged at 37°C. Scale bar, 5 μm.

Further, we examined whether the DNB aptamer within a tRNA scaffold could be used as a genetically encoded tag to image an RNA of interest in live bacterial cells with different fluorogenic probes. *Escherichia coli* bacteria were transformed with a plasmid expressing the DNB aptamer embedded within the tRNA scaffold (pET28-DNB) (31). As a negative control, we also transformed a plasmid expressing only the tRNA scaffold (pET28-tRNA). We observed a significant fluorescence signal in the bacterium expressing the DNB aptamer after 5 minutes of incubation with either RG-DN, TMR-DN or SR-DN (1 μM each) at 37°C, whereas no signal was observed in bacteria transformed with the control plasmid (Figure 5). Depending on the chosen probe, green (RG-DN), orange/red (SR-DN), or yellow (TMR-DN) fluorescence was observed. Since the probes become fluorescent only when bound to the RNA, no washing steps are required to image RNA in bacteria. Autofluorescence due to the metabolites inside the bacteria (in the absence of the fluorogenic probes) is more than two orders of magnitude lower compared to the signal obtained from DNB expressing bacteria incubated with SR-DN, TMR-DN or RG-DN. Next, we quantified the fluorescence values inside the bacteria expressing the DNB aptamer or the tRNA scaffold in the presence of the fluorogenic probes. The bacteria expressing DNB aptamer had ∼40-, ∼20- and ∼6-fold higher fluorescence than the bacteria expressing tRNA scaffold after incubation with TMR-DN, SR-DN and RG-DN, respectively. We also carried out time lapse experiments to find the optimum labelling time in live bacteria. The fluorescence inside the bacteria was significantly higher than the background already after 1 min of incubation, and the fluorescence reached maximum values within ∼10 min for TMR-DN and ∼2 min for RG-DN (Supplementary Figures S5 and S6). A probe concentration of 1 μM was sufficient for all imaging purposes. Hence, we could specifically label RNA in bacterial cells with cell-permeable dyes possessing different spectroscopic properties. This result highlights the advantage of using a contact quencher-binding aptamer over the fluorophore-binding aptamer as it provides the freedom to change the fluorophore according to the needs of the particular experiment.

### Orthogonal imaging of DNB and SRB-2 in bacterial cells

**Figure 6.**
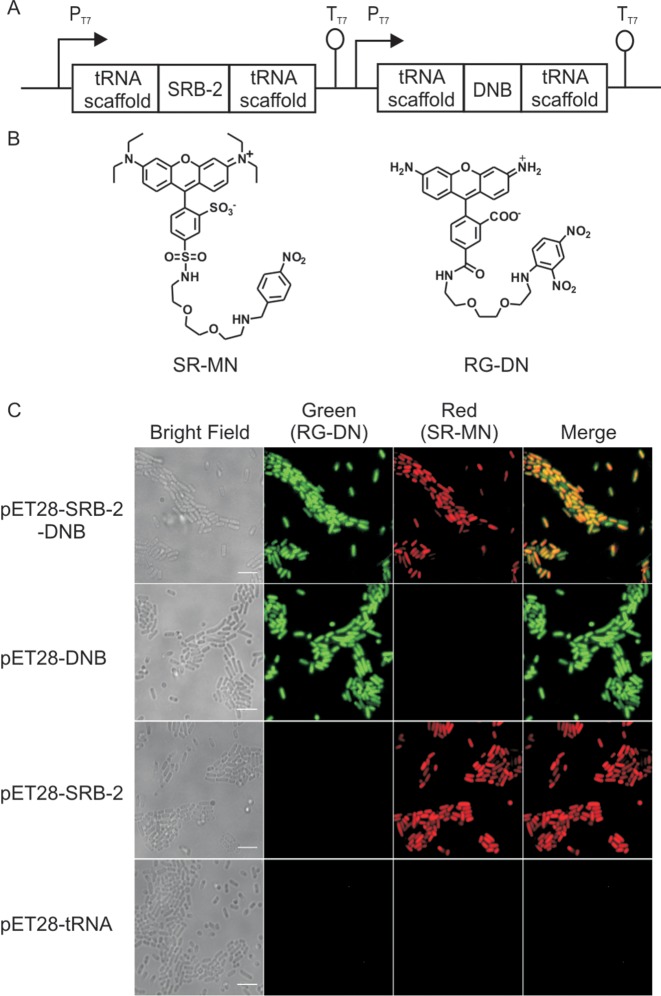
Dual colour labelling strategy using a quencher-binding aptamer and a fluorophore-binding aptamer. (**A**) Cloning vector used for expressing both SRB-2 and DNB (pET-SRB-2-DNB) aptamers in *E. coli*. (**B**) Structure of the two fluorogenic probes used for dual-colour imaging, SR-MN: sulforhodamine B-*p*-nitrobenzyl­amine and RG-DN: Rhodamine green-dinitroaniline. (**C**) Imaging DNB and SRB-2 aptamers in live *E. coli* with RG-DN and SR-MN (1 μM, each), respectively. Fluorescence signal in both red and green channel was detected in cells expressing both DNB and SRB-2, while cells expressing either DNB or SRB-2 showed fluorescence only in the green or red channel, respectively. Cells expressing the tRNA scaffold showed no fluorescence signal. Scale bar, 5 μm.

Finally, we designed a labelling strategy for simultaneous imaging of two distinct RNAs in bacterial cells by using the quencher-binding DNB aptamer and the fluorophore-binding SRB-2 aptamer (16) (sulforhodamine B binding aptamer) (Figure 6A, Supplementary Figure S7). Previously, we showed that the SRB-2 aptamer could be used to image RNA in live bacterial cells using the SR-DN probe (22). However, the combination of the DNB/SRB-2 aptamer pair with the RG-DN (green)/SR-DN (red) probe pair would not allow dual-colour imaging of two different RNA molecules since the DNB aptamer would bind to both probes. Therefore, instead of using dinitroaniline as quencher for sulforhodamine B, we conjugated another contact quencher, namely *p*-nitrobenzyl­amine (MN), to yield the probe SR-MN for exclusive labelling of the SRB-2 aptamer (Figure 6B). To examine our design, we constructed a plasmid (pET28-SRB-2-DNB) which allows for the transcription of both SRB-2 and DNB aptamers in a single bacterium under an independent promoter system (Figure 6A). Bacteria carrying the pET28-SRB-2-DNB plasmid were grown in LB medium and transcription was induced by addition of IPTG. After 5 minutes incubation of the bacteria with SR-MN and RG-DN, fluorescence signals for both probes were observed in a single bacterium, confirming the expression and correct folding of both aptameric tags. Further, the orthogonality of the system was confirmed as the fluorescence was observed only when the cognate pair of aptamer and probe (DNB/RG-DN and SRB-2/SR-MN) was present (Figure 6C, Supplementary Figure S8). Together, these data indicate the suitability of our approach for the simultaneous imaging of two RNAs in live bacteria.

## DISCUSSION

In summary, we presented a multi-faceted method for imaging RNA in living cells using a spectrum of fluorophore-quencher conjugates that cover a large part of the visible spectrum. To this end, we developed a new class of dinitroaniline conjugated fluorogenic probes which light up upon binding to a specific RNA aptamer. All of the synthesized probes (FL-DN, RG-DN, TMR-DN, SR-DN and TR-DN) were significantly quenched via contact quenching and showed very low fluorescence quantum yields in aqueous solution. However, in the presence of the quencher binding aptamer (DNB), fluorescence increased dramatically due to the disruption of the non-fluorescent complex formed between the fluorophore and dinitroaniline. The use of a high-affinity, specific quencher-binding aptamer (DNB) to restore the fluorescence of a palette of fluorogenic probes provides the important opportunity to select an appropriate fluorophore for the specific requirements of an experiment.

Quencher binding aptamers were previously used for detection of RNA *in vitro*, however the mechanisms of quenching were significantly different from the one used here. Murata *et al*. developed a method for real-time detection of mRNA transcripts *in vitro* (24) by using a black hole quencher (BHQ1), which acts through a combination of both FRET and contact quenching. Very recently, BHQ1 aptamer was engineered to form a specific RNA targeting aptamer (RT-aptamer) by replacing the original stem with two short RNA sequences complementary to 24-base sequence present in a target RNA such that the BHQ1-recognition loop is only formed when the RT-aptamer hybridizes with the respective target RNA (33). The specific RT-aptamer allowed imaging of several endogenous mRNAs. However, as this approach is based on hybridization, many mRNAs could not be detected due to inaccessibility of target sites *in vivo*. In addition, Sparano et al. developed aptamers against a derivative of aniline, which quenches the fluorescence of 2′,7′-dichlorofluorescein via photoinduced electron transfer (PeT) (25). Both FRET and PeT based approaches yielded quite moderate fluorescence enhancement factors (∼5–6-fold) upon binding of the probes to the respective quencher binding aptamers. In our contact quenching-based approach, we observed up to ∼70-fold fluorescence enhancement. Dinitroaniline is not an efficient FRET quencher for the fluorophores used in this study, as its absorption spectrum does not significantly overlap with the emission spectrum of the fluorophores and it cannot behave as a good PeT quencher due to the long ethylene glycol linker.

We furthermore demonstrated that this strategy in combination with a fluorophore-binding aptamer (SRB-2) could also be used for dual-colour imaging of RNA inside live bacteria, thereby facilitating research on RNA–RNA interactions and co-localization of RNAs in a cellular environment. Importantly, the DNB/DN probe combination is not only fully orthogonal to the SRB-2 system, but also to many other commonly used RNA imaging methods such as MS2-GFP or the Spinach system. Therefore, it would allow for efficient multiplexing as spectral bleed-through can be minimized by choosing an appropriate fluorophore with orthogonal emission properties. Previously, it was shown that, despite lacking organelles, bacterial cells can spatially organize not only proteins but also RNAs such that the cell interior is functionally compartmentalized (34,35). Thus, our work provides new tools that facilitate the investigation of RNA localization in bacterial cells as they enable the synchronous labelling of multiple RNAs using small molecules without the need for tedious washing procedures.

Furthermore, it is noteworthy to state that DNB aptamer is able to fold properly in the presence of biologically relevant magnesium concentrations and its melting temperature is significantly higher than the physiological temperature. Therefore, it is a very promising tag for *in vivo* imaging of target RNAs. In addition, TMR-DN and SR-DN probes when bound to DNB are about ∼2-fold brighter than GFP (Table 1), and they might allow for single-molecule imaging of target RNAs fused to DNB *in vivo*.

In conclusion, our strategy enables imaging of highly abundant target RNAs in live bacterial cells. However, imaging RNAs that are scarcely present in the cells requires further improvement of the methodology to increase sensitivity. Tandem repeats of RNA tags have been previously used to image low-abundance RNAs in cells successfully (36–38). Combining this strategy with the DNB aptamer we are currently working towards imaging of rarely represented RNA species in cells.

## Supplementary Material

SUPPLEMENTARY DATA
