# Supplementary material for: Dual-colour imaging of RNAs using quencher- and fluorophore-binding aptamers
Source: Nucleic Acids Res. 2015 Jul 14;43(21):e144. doi: 10.1093/nar/gkv718 (PMC4666381; doi:10.1093/nar/gkv718)
Supplement: SUPPLEMENTARY DATA [file supp_43_21_e144__index.html]

Dual-colour imaging of RNAs using quencher- and fluorophore-binding aptamers — Dual-colour imaging of RNAs using quencher- and fluorophore-binding aptamers — SUPPLEMENTARY DATA 

# Dual-colour imaging of RNAs using quencher- and fluorophore-binding aptamers

## SUPPLEMENTARY DATA

- SUPPLEMENTARY DATA
