# Supplementary material for: Dual-colour imaging of RNAs using quencher- and fluorophore-binding aptamers
Source: Nucleic Acids Res. 2015 Jul 14;43(21):e144. doi: 10.1093/nar/gkv718 (PMC4666381; doi:10.1093/nar/gkv718)
Supplement: SUPPLEMENTARY DATA [file supp_gkv718_nar-01449-met-g-2015-File008.pdf]

Supplementary Information

Dual-colour imaging of RNAs using quencher- and fluorophore-binding aptamers

Ankita Arora[†], Murat Sunbul[†], Andres Jäschke

Institute of Pharmacy and Molecular Biotechnology, Heidelberg University, 69120 Heidelberg, Germany.

Correspondence should be addressed to: jaeschke@uni-hd.de

[†] These authors contributed equally to this work.

A) Supplementary Material and Methods

1. Preparation of the affinity resin

NHS-activated sepharose (GE Life Sciences) was washed with 2 volumes of ice-cold Millipore water. The resin was resuspended in 100 mM HEPES buffer (pH 7.4). Dinitroaniline-PEG_3-Amine (12.1 mg, 38.5 µmol, for synthesis see section B1) dissolved in 500 µL of DMSO was added into the resin dropwise with vigorous shaking to ensure homogenous functionalization of the resin, and then incubated at 25°C for 3 hours. Further, the resin was incubated with 0.5 M ethanolamine at 25°C for 2 h to react with any free NHS-activated sites. The resin was washed thoroughly and stored in 100 mM Tris buffer (pH 7.5) at 4°C. The coupling efficiency was determined by measuring the absorbance of unreacted dinitroaniline in the flow-through. Using this strategy, we estimated that the resin contained 7 µmol of quencher per ml of resin. Mock-resin was also prepared by using the same approach where only DMSO was added to the resin instead of Dinitroaniline-PEG_3-Amine.

2. Library design and preparation

To prepare a partially structured RNA library, a DNA oligonucleotide was synthesized that contained two fixed primer binding sites flanking a 64-nucleotide region. This region consisted of two 26-base random stretches that were separated by a 12-base constant region designed to form a stable CCGU stem-loop in transcribed RNA (Supplementary Figure S1B). The single-stranded oligonucleotide was synthesized in 1 µmol scale and phosphoramidites for the random regions were mixed in a ratio of 3:3:2:2 (A:C:G:T). The randomized single-stranded oligonucleotide was PAGE-purified, and 1.6 nmol of the oligonucleotide were amplified in a 30 mL PCR reaction for 6-cycles by using the forward and reverse primers (see Supplementary Table S3 for sequences) to yield double-stranded DNA template for transcription of the library. The PCR product was precipitated with sodium acetate and ethanol after phenol:chloroform:isoamyl alcohol (25:24:1) extraction. The DNA pellet was dissolved in water and directly used for *in vitro* transcription reaction.

3. Random mutagenesis of round 15 pool (first SELEX) and clone 5 (best aptamer identified from round 15)

Random mutagenesis was performed by a mutagenic PCR protocol (1), that was developed to increase mutation frequencies involving high Mg concentration, unequal dNTPs ratios, and addition of $MnCl_2$. For PCR, the following reagents were added: 1X PCR buffer (Rapidozyme; 67 mM Tris, *p*H 8.8, 16 mM $(NH_4)_2SO_4$, 0.01% Tween 20), 7 mM $MgCl_2$, 0.3 µM of forward primer and 0.3 µM of reverse primer, 0.2 mM dGTP, 0.2 mM dATP, 1 mM dCTP, 1 mM dTTP, 0.5 mM $MnCl_{2,}$ and 0.05 U/µL Taq polymerase (Rapidozyme) to 200 pg of template per reaction. DNA was amplified under the following conditions: 94°C/2 min, 25 cycles of {94°C/1 min, 52°C/1 min, 72°C/1 min}, 72°C/20 min. The PCR product was analysed on a 2 % agarose gel (with ethidium bromide, using standard electrophoresis conditions; 1X TBE, 120 V, 40 min). The PCR product was used as a template for the next cycle of mutagenesis using the same conditions as before. The PCR product from the second cycle of mutagenesis was cloned and sequenced to validate the frequency of mutations incorporated. Sequencing results showed an average of 2 mutations per sequence. Mutated round 15 pool and mutated clone 5 obtained by random mutagenesis were mixed in a ratio of 7:3 and used as a pool for the second *in vitro* selection (SELEX).

4. Structure predictions and truncation studies

The RNA sequences obtained from sequencing the pool were subjected to secondary structure prediction using Mfold software (2). The active clones were truncated by deleting the constant primer binding regions from both ends. Mutated and truncated aptamers were created by ordering single-stranded DNA templates (Integrated DNA Technologies) with desired mutations and PCR-amplified to form double-stranded templates. PCR products were purified with a PCR purification kit (QiaGen) and used as templates for *in vitro* T7 transcription reactions.

B) Synthesis of the compounds

1. Synthesis of Dinitroaniline-PEG3-Amine (DN-PEG3-Amine)

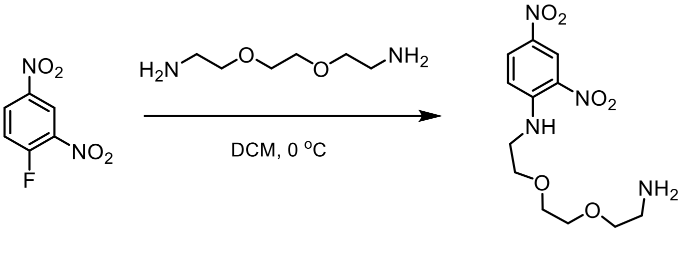

DN-PEG$_3$-Amine

To a stirring solution of 2,2'-(ethylenedioxy) bisethylenediamine (3.98 g, 27 mmol) in 20 mL of dichloromethane (DCM) at 0°C was added a solution of dinitrofluorobenzene (0.50 g, 2.7 mmol) in 10 mL of DCM, dropwise. After the addition was complete, the temperature was brought to room temperature and the mixture was stirred for 30 minutes. Then, the reaction mixture was mixed with 100 mL of water and the organic phase was recovered. The organic phase was mixed with 100 mL of 0.1 M HCl and the product was taken into the acidic aqueous phase and the DCM phase was discarded. Finally, the pH of the aqueous phase was adjusted to 12-13 with NaOH and the product was extracted into the DCM phase using 2 x 100 mL of solvent. Organic phases were combined, washed with brine (100 mL), dried over Na_2SO_4, filtered and evaporated to yield DN-PEG3-Amine (0.77 g, 91%). The product was used without further purification for the next step. HR-ESI (positive): calculated 315.1299, found 315.1310 for $C_{12}H_{19}N_4O_6$.

2. Synthesis of Dinitroaniline-PEG3-SS-PEG4-Biotin (DN-SS-Biotin)

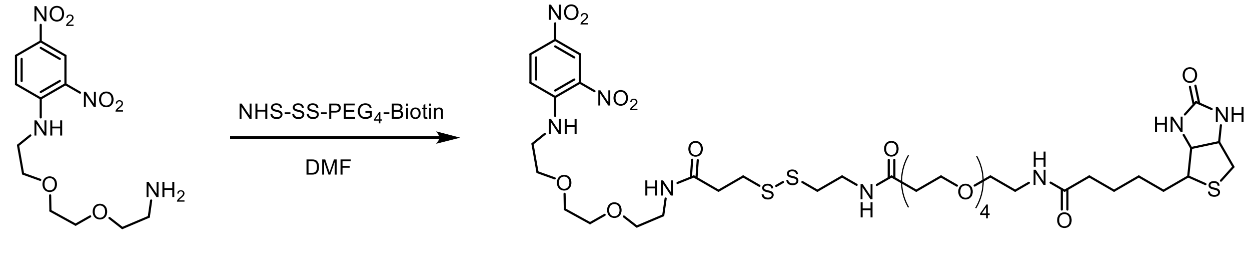

DN-SS-Biotin

To a mixture of DN-PEG$_3$-Amine (3.5 mg, 11.1 µmol), EZ-Link NHS-SS-PEG$_4$-Biotin (6.3 mg, 8.4 µmol) in 400 µL of anhydrous dimethylformamide (DMF) was added triethylamine (1.1 µL, 7.9 µmol) and the reaction was incubated at 30°C for 30 minutes. The product was purified by reverse-phase HPLC (40% acetonitrile, 0.1% TFA) to yield DN-SS-Biotin (3.4 mg, 43%). HR-ESI (positive): calculated 973.3440, found 973.3421 for $C_{38}H_{62}N_8O_{14}S_3Na$.

3. Synthesis of Fluoroscein-PEG3-dinitroaniline (FL-DN)

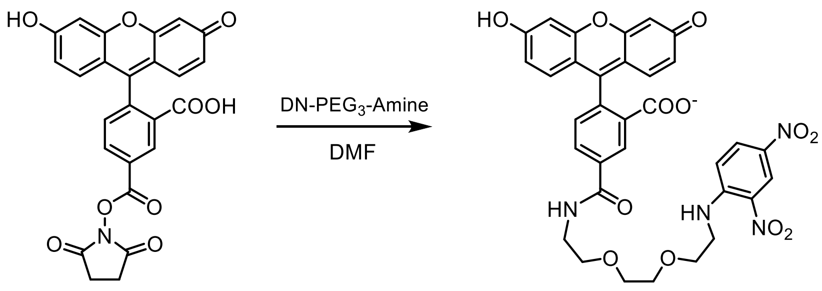

FL-DN

To a solution of 5-carboxy-fluorescein-N-hydroxysuccinimide (1.0 mg, 2.1 μmol) in DMF (100 μL) was added a solution of DN-PEG3-Amine (2.0 mg, 6.3 μmol) dissolved in DMF (100 μL) and the reaction mixture was stirred at room temperature for 15 min. The reaction mixture was purified on a reverse phase C-18 column (50% acetonitrile, 0.1% trifluoracetic acid) to yield the TFA salt of FL-DN (0.5 mg, 35%). HR-ESI (positive): calculated 695.1596, found 695.1595 for $C_{33}H_{28}N_4O_{12}Na$.

4. Synthesis of Rhodamine green-PEG3-dinitroaniline (RG-DN)

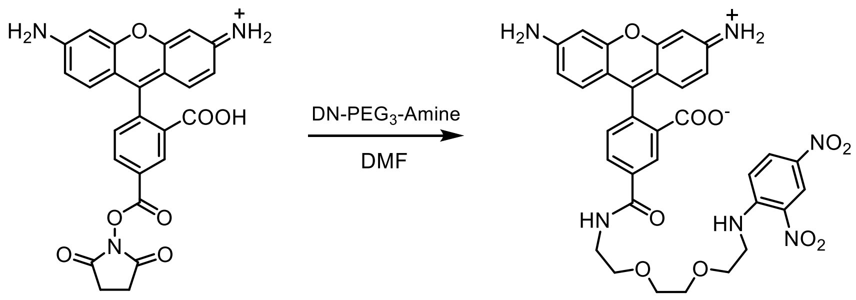

RG-DN

To a solution of 5(6)-carboxy-rhodamine 110-N-hydroxysuccinimide (2.9 mg, 5.7 μmol) in 100 μL of DMF was added a solution of DN-PEG3-Amine (5.4 mg, 17.2 μmol) dissolved in 100 μL of DMF and the reaction mixture was stirred at room temperature for 15 minutes. The reaction mixture was purified on a reverse phase C-18 column (50% acetonitrile, 0.1% trifluoracetic acid) to yield RG-DN (2.1 mg, 55% yield). HR-ESI (positive): calculated 671.2096, found 671.2122 for $C_{33}H_{31}N_6O_{10}$.

5. Synthesis of Tetramethylrhodamine-PEG3-dinitroaniline (TMR-DN)

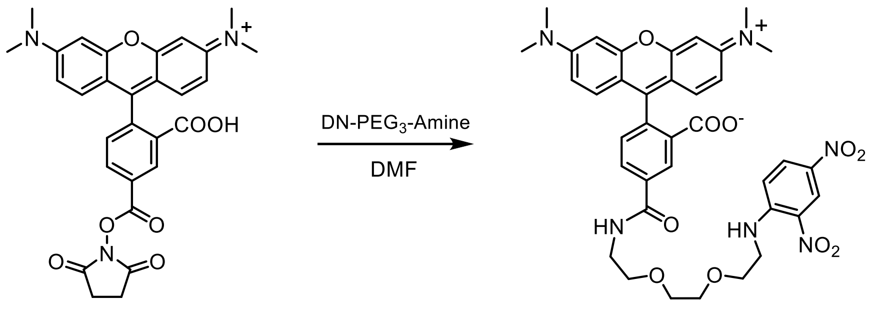

TMR-DN

To a solution of 5-carboxy-tetramethylrhodamine-N-hydroxysuccinimide (1.0 mg, 1.9 μmol) in 100 μL of DMF was added a solution of DN-PEG3-Amine (1.8 mg, 5.7 μmol) dissolved in 50 μL of DMF and the reaction mixture was stirred at room temperature for 15 min. The reaction mixture was purified on a reverse phase C-18 column (60% acetonitrile, 0.1% trifluoracetic acid) to yield TMR-DN (1.0 mg, 72% yield). HR-ESI (positive): calculated 727.2722, found 727.2700 for $C_{37}H_{39}N_6O_{10}$.

6. Synthesis of Sulforhodamine B-PEG3-dinitroaniline (SR-DN)

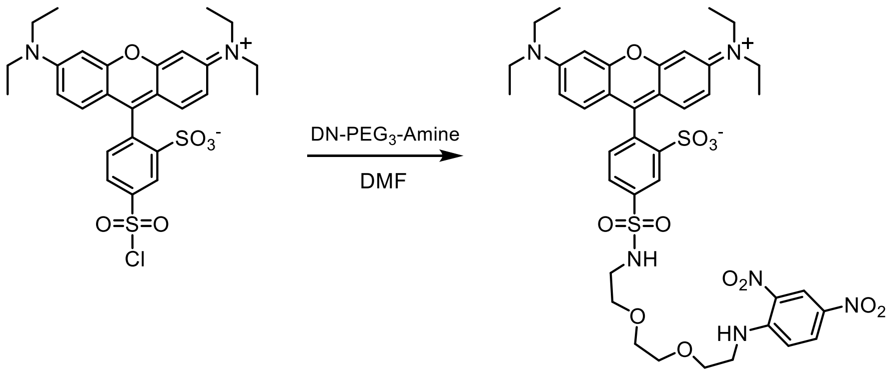

SR-DN

To a solution of sulforhodamine B acid chloride (10 mg, 17 μmol) in 200 μL of DMF was added a solution of DN-PEG3-Amine (16 mg, 51 μmol) dissolved in 200 μL of DMF and the reaction mixture was stirred at room temperature for 15 min. The reaction mixture was purified on a reverse phase C-18 column (50% acetonitrile, 0.1% trifluoracetic acid) to yield SR-DN (5.2 mg, 35% yield). HR-ESI (positive): calculated 877.2507, found 877.2496 for $C_{39}H_{46}N_6O_{12}S_2Na$.

7. Synthesis of TexasRed-PEG3-dinitroaniline (TR-DN)

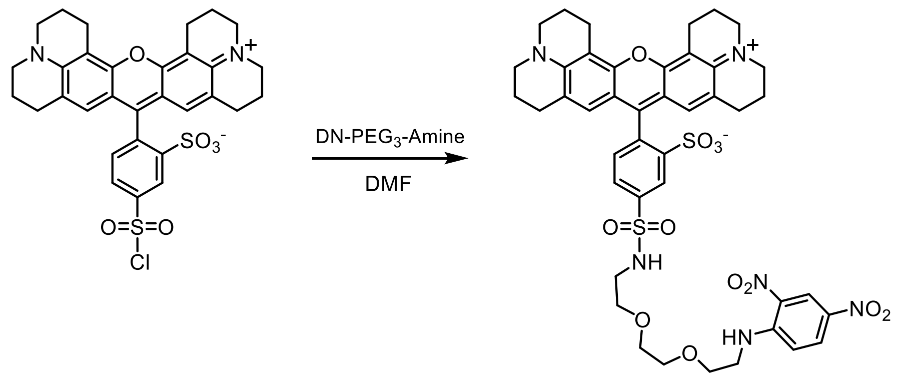

TR-DN

To a solution of TexasRed-sulfonylchloride (1.9 mg, 3.0 µmol) in 100 µL of DMF was added a solution of DN-PEG3-Amine (2.9 mg, 9.0 µmol) dissolved in 100 µL of DMF and the reaction mixture was stirred at room temperature for 15 min. The reaction mixture was purified on a reverse phase C-18 column (50% acetonitrile, 0.1% trifluoracetic acid) to yield TR-DN (1.1 mg, 40% yield). HR-ESI (positive): calculated 925.2507, found 925.2520 for $C_{43}H_{46}N_6O_{12}S_2Na$.

C) Supplementary Figures

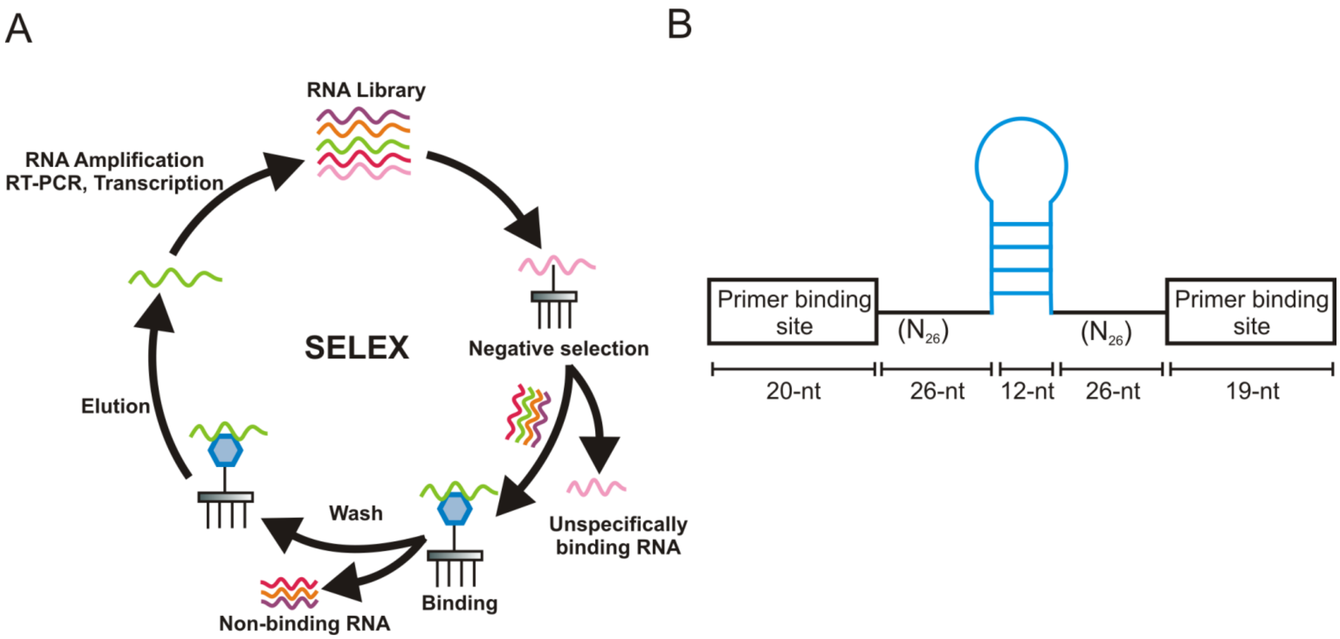

Supplementary Figure S1. Systematic evolution of ligands by exponential enrichment (SELEX) (A) Schematic of the SELEX to select RNA that binds to dinitroaniline. (B) Design of the partially structured RNA library comprising of two fixed primer binding sites flanking a 64-nucelotide region consisting of two 26-base random region inter-spersed by a 12- base constant region forming a stable stem-loop (blue). Such partially structured libraries have been reported to increase the probability of evolving high-affinity binders (3).

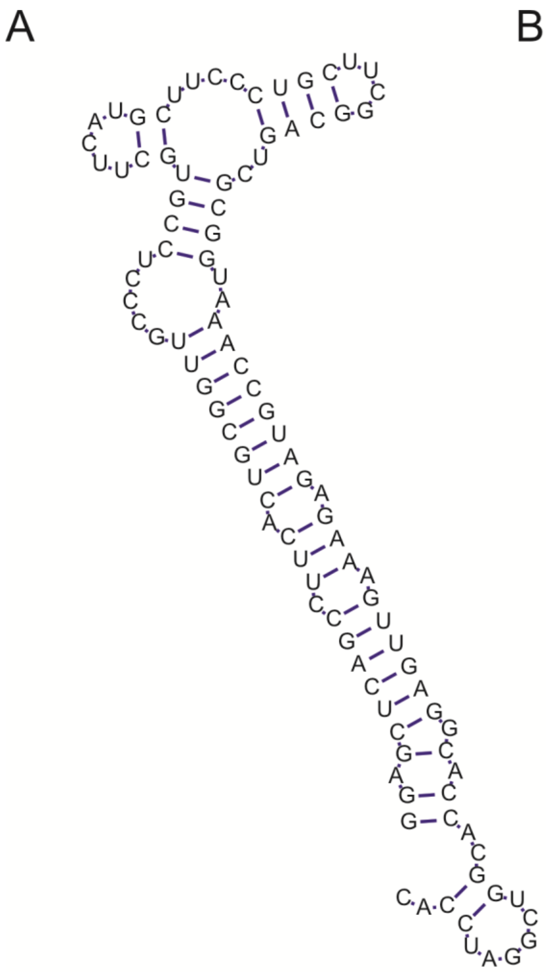

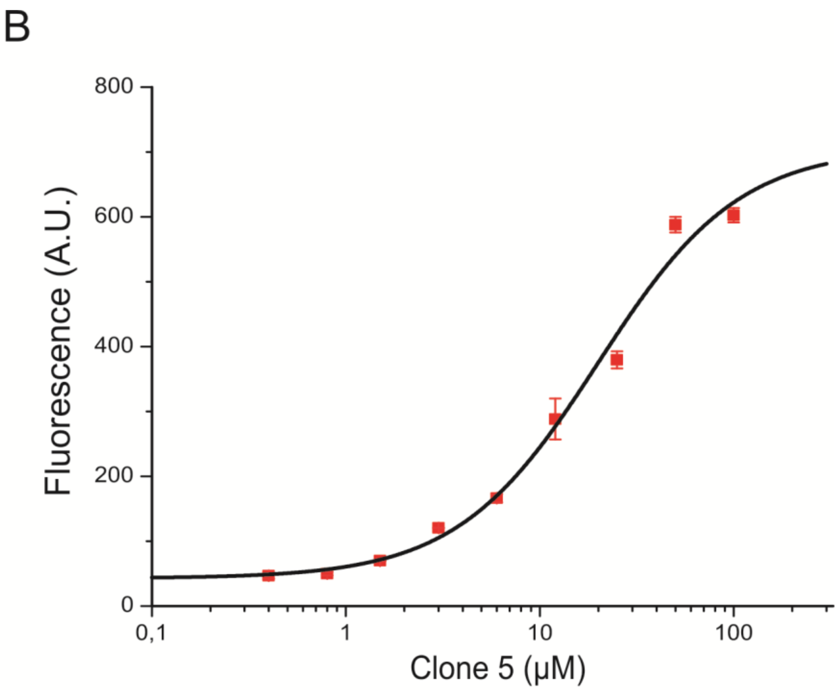

Probe	Fold enhancement	K_D (μM)
FL-DN	4	27.2±3.2
RG-DN	N.D.	N.D.
TMR-DN	8	7.77±0.9
SR-DN	10	20.1±3.9
TR-DN	12	52.6±17.8

Supplementary Figure S2. Characterization of the clone 5 identified from round 15 of the first SELEX. (A) Mfold-predicted secondary structure of clone 5. (B) Dissociation constant (K_D) of clone 5 – SR-DN complex. K_D was determined by measuring the fluorescence increase upon addition of the aptamer to SR-DN probe (1 μM) and it was calculated as 20.1±3.9 μM. K_D between the aptamer and other probes (FL-DN, TMR-DN and TR-DN) were measured similarly and were found to be 27.2±3.2 μM, 7.77±0.9 μM and 52.6±17.8 μM for FL-DN, TMR-DN and TR-DN, respectively.

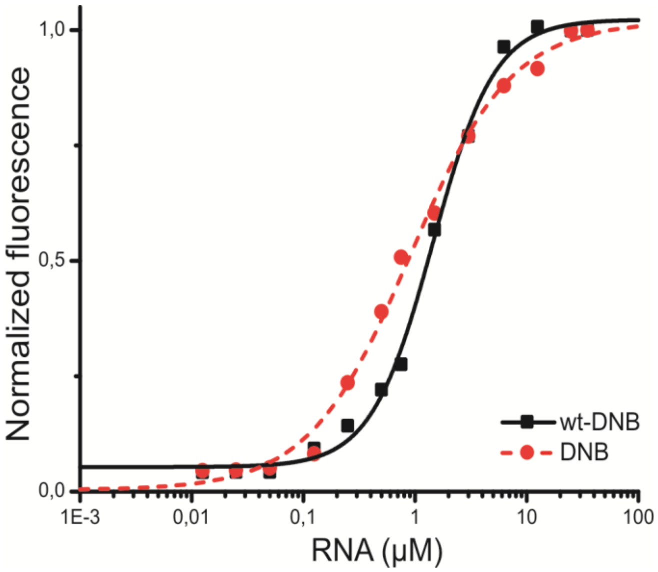

Supplementary Figure S3. Determination of the dissociation constant (K_D) between SR-DN and wt-DNB (black curve) or DNB (red curve). K_D values were calculated by measuring the increase in fluorescence upon addition of the aptamer, keeping the SR-DN concentration constant (100 nM). The K_D values were found to be 1.44±0.08 µM and 0.80±0.1 µM for wt-DNB and DNB, respectively.

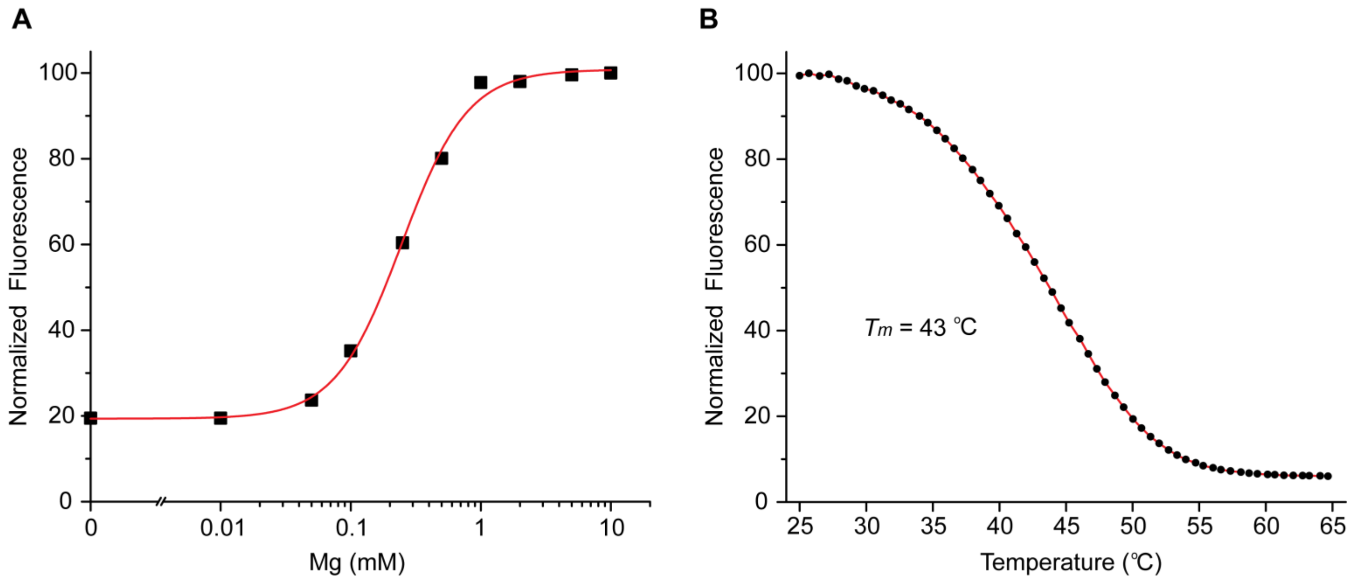

Supplementary Figure S4. Characterization of DNB aptamer. (A) Magnesium dependence of DNB aptamer. To measure magnesium dependence, 1 μM of DNB aptamer was folded in the presence of different amount of magnesium ions (0-10 mM) and the fluorescence values were recorded upon addition of 1 μM of TMR-DN probe. (B) Temperature dependence of the DNB aptamer. 1 μM of the DNB aptamer was incubated with 1 μM of TMR-DN and fluorescence decay was followed upon increasing the temperature from 25°C to 65°C. T_m was determined to be 43°C.

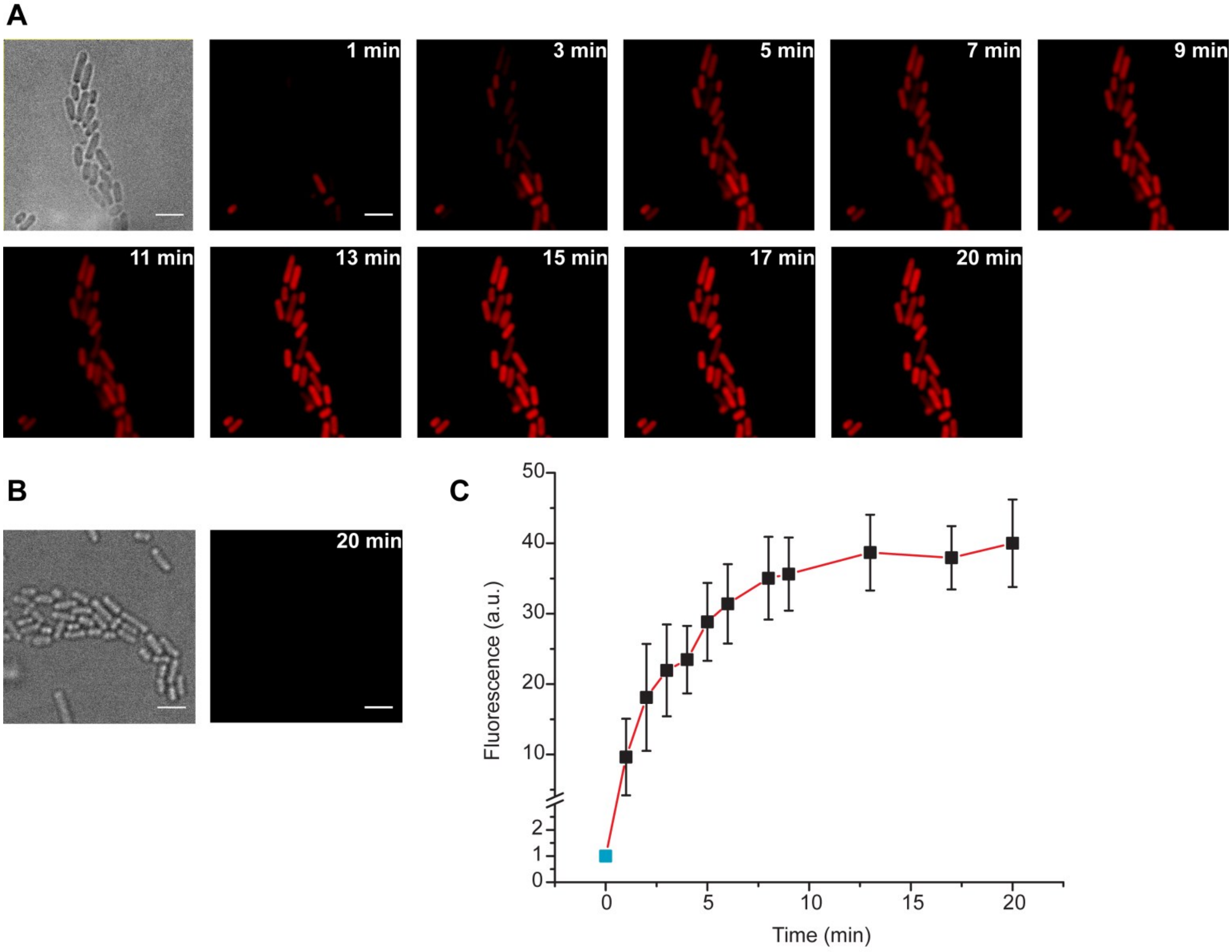

Supplementary Figure S5: Time lapse experiments to determine the optimal incubation time for labelling RNA with TMR-DN. (A) Bacterial cells were transformed with pET28-DNB plasmid and transcription was induced with 1 mM IPTG. 1 μM of TMR-DN was added and fluorescence images were taken every minute for 20 minutes. No further increase in fluorescence signal was observed after 10 minutes of incubation. (B) As a negative control, bacterial cells were transformed with pET28-tRNA and expression was induced with 1 mM IPTG. No fluorescence signal was observed in cells expressing the tRNA scaffold after incubation with 1 μM of TMR-DN for 20 min. (C) The fluorescence signal from single bacterial cells was quantified using Image J and plotted over time. Blue square (at t= 0 min) indicates the background fluorescence in control bacteria and it remains constant during the course of the measurement. Error bars indicate standard deviations (n=32). Scale bar, 3 μm.

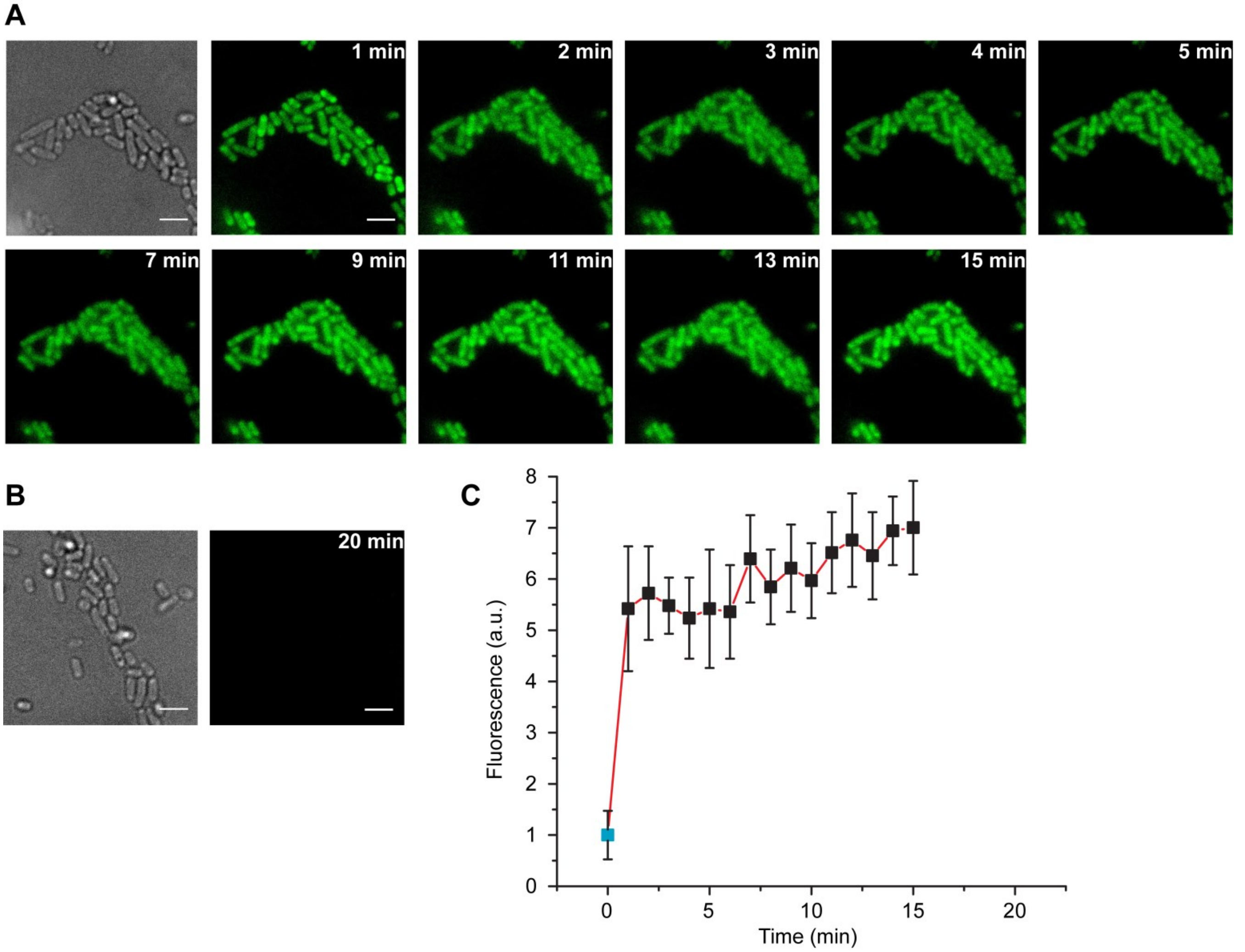

Supplementary Figure S6: Time lapse experiment to determine the optimal incubation time for labelling RNA with RG-DN. (A) Bacterial cells were transformed with pET28-DNB plasmid and transcription was induced with 1 mM IPTG. 1 μM of RG-DN was added and fluorescence images were taken every minute for 15 minutes. No further increase in fluorescence signal was observed after 2 minutes of incubation. (B) As a negative control, bacterial cells were transformed with pET28-tRNA and expression was induced with 1 mM IPTG. No fluorescence signal was observed in cells expressing the tRNA scaffold after incubation with 1 μM of RG-DN for 20 min. (C) The fluorescence signal from single bacterial cells was quantified using Image J and plotted over time. Blue square (at t= 0 min) indicates the background fluorescence in control bacteria and it remains constant during the course of the measurement. Error bars indicate standard deviations (n=32). Scale bar, 3 μm.

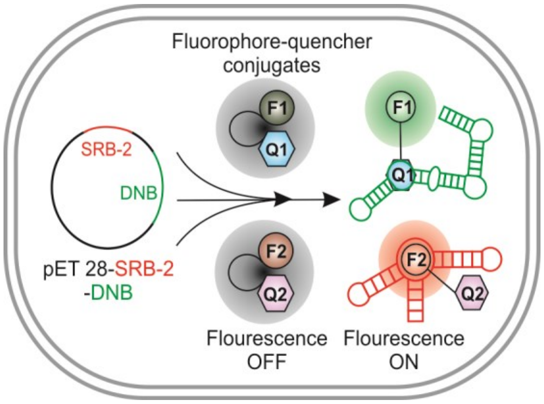

Supplementary Figure S7. Schematic of the design for dual-colour imaging of different RNAs in living bacterial cells. F1 and F2 denote fluorophores and Q1 and Q2 denote contact quenchers. Q1 binds to a quencher binding aptamer and F2 binds to a fluorophore binding aptamer. The dinitroaniline-binding RNA (DNB, quencher-binding aptamer) and sulforhodamine B-binding RNA (SRB-2, fluorophore-binding aptamer) were expressed in *E. coli* and incubated with two light-up probes (SR-MN and RG-DN) carrying different quenchers. Upon expression of the RNAs, the analogous pair of the aptamer and the probe forms a complex (SR-MN/SRB-2 and RG-DN/DNB) and results in a fluorescence signal.

Bright Field | Green (RG-DN + DNB) | Red (SR-MN + SRB-2) | Merge

pET28-SRB-2-DNB +RG-DN

pET28-SRB-2-DNB +SR-MN

pET28-DNB +SR-MN

pET28-SRB-2 +RG-DN

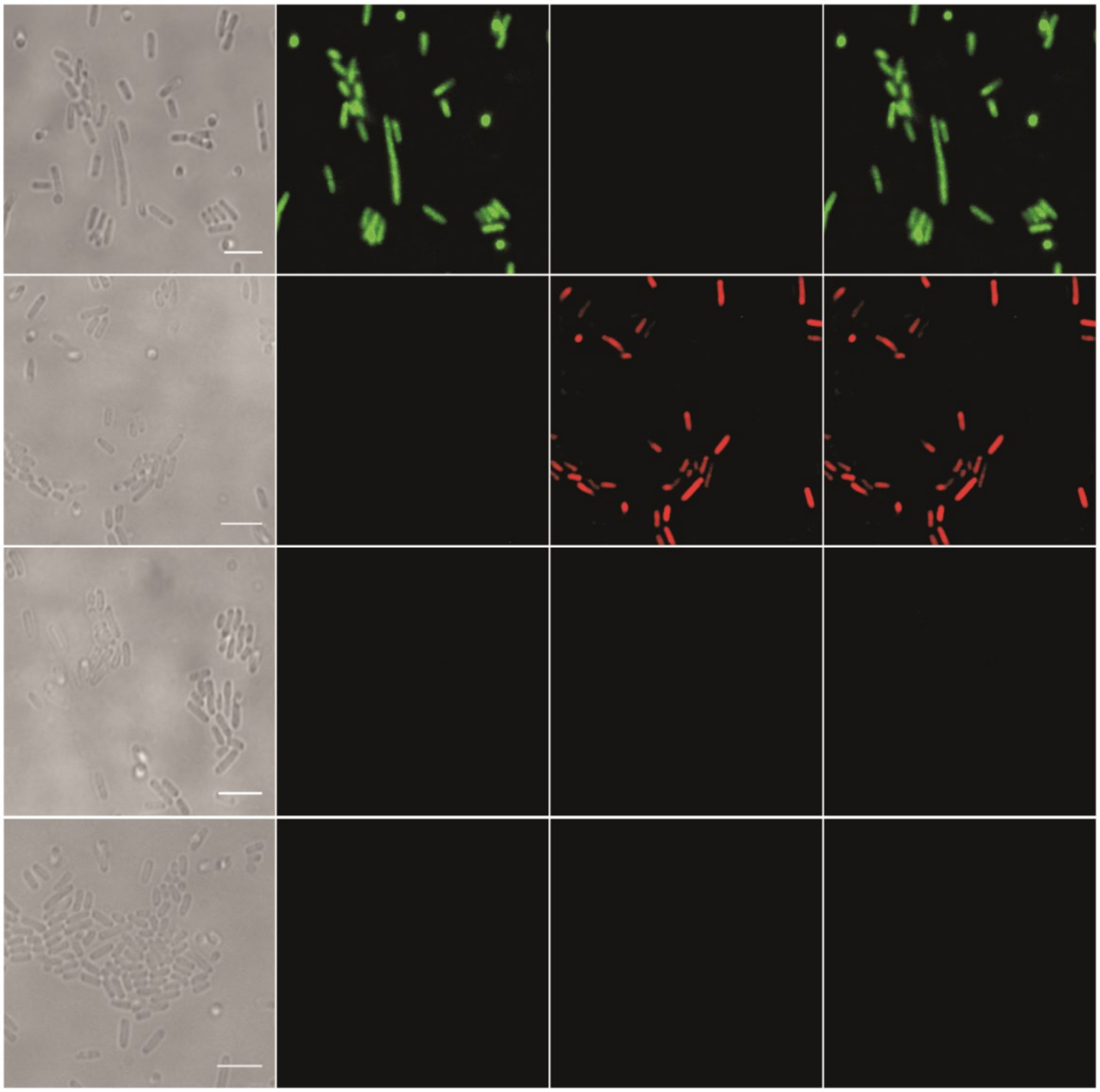

Supplementary Figure S8. Orthogonality of SRB-2 and DNB tags in *E. coli*. Bacteria expressing both DNB and SRB-2 aptamers were incubated with either 1 μM of RG-DN or 1 μM of SR-MN and they showed fluorescence only in the green channel (first row) or only in the red channel (second row), respectively. No fluorescence signal was observed in bacteria expressing only DNB aptamer after incubation with 1 μM of SR-MN (third row), and similarly no fluorescence signal was observed in bacteria expressing only SRB-2 aptamer after incubation with 1 μM of RG-DN (fourth row). These data confirm that there is no cross-binding between the pair of aptamers and probes used. Scale bar, 5 μm.

D) Supplementary Tables

Supplementary Table S1: Sequences of the colonies picked from round 15 pool of SELEX 1

Colony number	RNA sequence	Abundance/52
>9	GGAGCUCAGCCUUCACUGCACAACGCCCUUGUGCAAAACUCUCGCCUGCUCGGCAGGU<br>UUACCGAUAGGACGAUCGGUGGAAGGCACCACGGUCGGAUCCAC	4
>1	GGAGCUCAGCCUUCACUGCUGACGAAAAAACCGUGAACGGAAUAUCUGCUUCGGCAGUG<br>UCGGCGUGAUGAUGGGUUCAACCUGGCACCACGGUCGGAUCCAC	3
>6	GGAGCUCAGCCUUCACUGCACACAGAUUAAAUAGCAAACAUAAAUCUGCUUCGGCAGCU<br>GUGUUACCGUUGCCAGUAAGCUAUGGCACCACGGUCGGAUCCAC	3
>2	GGAGCUCAGCCUUCACUGCCGUGUGCUCCGCGGGCCGAACGCCGUCUGCUUCGGCAGU<br>GUUGAACCUUCGGAUCAGACCUUGAGGCACCACGGUCGGAUCCAC	2
>3	GGAGCUCAGCCUUCACUGCUAUAGCGUGCGCGAGUCAGUACGCUUCUGCUCCGGCAGC<br>CUGAAGACUUUUCUGACCGAUGACAGGCACCACGGUCGGAUCCAC	2
>10	GGAGCUCAGCCUUCACUGCCGUGUGCUCCGCGGGCCGAACGCCGUCUGCUGCGGCAGU<br>GUUGAACCUUCGGAUCAGACCUUGAGGCACCACGGUCGGAUCCAC	2
>13	GGAGCUCAGCCUUCACUGCACAACGCCCUUGUGCAAAACUCUCGCCUGCUCGGCAGGU<br>UUACCGAUAGGACGAUCGGUGGAAGGCACCACGGUCGGACCAC	2
>22	GGAGCUCAGCCUUCACUGCAGUGAAAAAAAUGUAAACAUAAGAUCUGCUUCGGCAGUCA<br>CUGCAUCGACAACACCGUGGGAUGGCACCACGGUCGGAUCCAC	2
>5	GGAGCUCAGCCUUCACUGCGGUUGCCCUCCGUGCUUCAUGCUUCCCUGCUUCGGCAGU<br>CGCGGUAAACCGUAGAGAAAGUUGAGGCACCACGGUCGGAUCCAC	1
>8	GGAGCUCAGCCUUCACUGCAGCAAAUAAAAAGCAAGCAUAUGAAUCUGCUUCGGAGGGA<br>CCCAAUGGAGCGUUGUGACCGUAGGCACCACGGUCGGAUCCAC	1
>14	GGAGCUCAGCCUUCACUGCUGGAGCCGGAAUUUCUGAGCAAUUCCCGCUUCGGCAGAG<br>UGGGUGAAUCCACUGUGGUCUGAAGGCACCACGGUCGGAUCCAC	1
>19	GGAGCUCAGCCUUCACUGCUUGCAUGCCGUUCAGGGAUUAUUACCCUGCUUGCAGUGA<br>CCGCGGGGCGAUCGCGUCCGCCAGGCACCACGGUCGGAUCCAC	1
>20	GGAGCUCAGCCUCACUCACAUGCAAACCUGAGAGUCAAGGAUUUGCUUCGGCAGUGAGU<br>CGACUGCGGUGCUGGUAAGCAGGCACCACGGUCGGAUCCAC	1
>27	GGAGCUCAGCCUUCACUGCGAUUGCGAACGAGAUUGCUGUGGCGGCUGCUCCGGCAGG<br>CUGACCCCGUGAUCCGUCCUUCGUAGGCACCACGGUCGGAUCCAC	1
>29	GGAGCUCAGCCUUCACUGCUGAAAAAAGUGCGAAUUGCAGCUGAAAACUGCUUCAGCAG<br>UGAAGUGCAUUAUGCCAACCGUGAUAGGCACCACGGUCGGAUCCAC	1
>37	GGAGCUCAGCCUUCACUGCUGGAGGCCAUGCUGUAGCCUCAGAACUGCUUCGGCAGCA<br>GUCCCGUUGGUGAGCCGAGGCCUGGGCCCACGGUCGGAUCCAC	1
>40	GGAGCUCAGCCUUCACUCUGGAGAUAUUCACACGACAAACAUUGCUGCUUCGGCAGAUC<br>CAGAUAGCCUUGGUGCGUAAGUCGGCACACGGUCGGAUCCAC	1
>41	GGAGCUCAGCCUUCACUGCUGAAAAGUGCGAAUUGCAGCUGAAAACUGCUUCGGCAGU<br>GAAGUGCAUUAUGCCAACCGUGAUAGGCACCACGGUCGGAUCCAC	1
>42	GGAGCUCAGCCUUCACUGCUGGGAUUAGCCAAGCGAGAAUCCCAUCUGCUUCGGCAGG<br>GUGAAGGAAUCCACUGUGGACGCAAGGCACCACGGUCGGAUCCAC	1
>43	GGAGCUCAGCCUUCACUGCAGUGAAAAAAUGUAAACAUAAGAUCUGCUUCGGCAGUCAC<br>UGCAUCGACAACACCGUGGGAAGGCACCACGGUCGGAUCCAC	1
>45	GGAGCUCAGCCUUCACUGCUUUGUAUCAAAAUACGCGGGAUUUUGCUGCUUCGGCAGG<br>UUUCCCCCGCGGGGAGACAUACAAGGCACCACGGUCGGAUCCAC	1
>46	GGAGCUCAGCCUUCACUGCUUUGUAUCAAAAUACGCGGGAUUUUGCUGCUUCGGCAGG<br>UCUCCCCCGCGGGGAGCACGUACAAGGCACCACGGUCGGAUCCAC	1
>47	GGAGCUCAGCCUUCACUGCGGCAAACAAAACAGUGAGGGCCUUUUCUGCUUCGCAGUG<br>UUGUAAGCCAGCACUGUAGAGGCCGGCACCACGGUCGGAUCCAC	1
>50	GGAGCUCAGCCUUCACUGCUUGCAAAUAUACAACAAAAAUCACUGCUUCGGCAAGACGU<br>GAAGUCACAGACCGACCGGUAUACCAGGCACCACGGUCGGAUCCAC	1
>54	GGAGCUCAGCCUUCACUGCUACGAUUUUGGUAAGCAAUUAUCGUGCUGCUCCGGCAGC<br>GACGGCUUCCGUGUGUCUGCAAACCGGCACCACGGUCGGAUCCAC	1
>56	GGAGCUCAGCCUUCACUGCCAGUCAACAGCAACAUGCCAAGCGAUCUGCUUUCGGCAGU<br>GACGACUCUUCAACCCCGUGGGCAAGGCACCACCGGUCGGAUCCAC	1
>57	GGAGCUCAGCCUUCACUGCGCCAGCCAAACAUGAAGCGCAUCAUACUGCUUCGGCAGAU<br>AGGCCGAUCCGGCUGAGUACGUAAGGCACCACGGUCGGAUCCAC	1
>4	GGAGCUCAGCCUUCACUGCCCGCUGCUAUCCUCCGCGAUAAAGACUGCUUCGGCAGGA<br>GAGCACCGCGACUCUCGAUCAUCGGGCACCACGGUCGGAUCCAC	1
>7	GGAGCUCAGCCUUCACUGCAUGCAUGCCGUUCAGGGAUUAUUACCCUGCUUGCAGUGA<br>CCGCGGGGCGAUCGCGUCCGCCAGGCACCACGGUCGGAUCCAC	1
>23	GGAGCUCAGCCUUCACUCAGGGCAUAGUAAGCGAUUAGAACACCUGCUUCGGCAAGCAA<br>ACUAUUGCCGUUAAGGAGCUCUGGCACCACGGUCGGAUCCAC	1

>30	GGAGCUCAGCCUCACUGCAGCUGGCAAAAUGCAGCUCACCUCUGUUUCGGCAGGGGAA AUACAUUAAGCGAUUCGUGUUGGCACCACGGUCGGAUCCAC	1
>32	GGAGCUCAGCCUUCACUGCUGAAAAAGUGCGAAUUGCAGCUGUAAACUGCUUCGGCAGU GAAGUGCAUUAUGCCAACCGUGAUAGGCACCACGGUCGGAUCCAC	1
>33	GGAGCUCAGCCUUCACUGCAGUGAUGAGCAACACCGGACCGACCUCUGCUUCGGCAGA CUGAACUGAGUGGGUAAGCACCUUCGGCACCACGGUCGGAUCCAC	1
>34	GGAGCUCAGCCUUCACUGCAGGGGGUUUUUUCGACGCCGCGAUCAACUGCUUCGGCAG GUCUAUAUCGACGUGUAGACGCCCCCGGCACCACGGUCGGAUCCAC	1
>36	GGAGCUCAGCCUUCACUGCUGAAAAGUGCGAAUUGCAGCUGGAAACUGCUUCGGCAGU GAAGUGCAUUAUGCCAACCGUGAUAGGCACCACGGUCGGAUCCAC	1
>38	GGAGCUCAGCCUUCAUGCUCGAUUAACCAUAAGUACAAAGAAUCUGCUUCGGCAGGAAU GGACUAUCCCGUGGUGACAAAUGGCACCACGGUCGGAUCCAC	1
>49	GGAGCUCAGCCUUCACUGCAGUGAAAAAAAUGUAAACAUAAGAUCUGCUUCGGCAGUCA CUGCAUCGACAACACCGUGGCACCACGGUCGGAUCCAC	1
>52	GGAGCUCAGCCUUCACUCAGGGCAUAGUAAGCGAUUAGAAUACCUGCUUCGGCAAGCAA ACUAUUGCCGUUAAGGAGCUCUGGCACCACGGUCGGAUCCAC	1
>53	GGAGCUCAGCCUUCACUGCUUGAGUUCCCGAAGUACGACAAGUCUGCUUCGGCAGGAA GGAACACCAAGGUGCGACAAGUCGGCACCACGGUCGGAUCCAC	1
>60	GGAGCUCAGCCUUCACUGCAGUGAAAAAAAUGUAAACAUAAGAUCUGCUUCGGCAGUCA CUGCAUCGACAACACCGUGGGACGGCACCACGGUCGGAUCCAC	1

Supplementary Table S2: Sequences of the colonies picked from round 9 pool of SELEX 2

Colony number	RNA sequence	Abundance/85
>C12	GGAGCUCAGCCUUCACUGCUAUAGCGUGCGCGAGUCAGUACGCUUCUGCUCCGGCAGC CUGAAGACUUUUCUGACCGAUGACAGGCACCACGGUCGGAUCCAC	3
>A03	GGAGCUCAGCCUUCACUGCGGCUAGGCUCCGCCGCGCGUGUACACUGCUUCGGCAGAC GACGUGUGAUUACGACGAAUCACUGGCACCACGGUCGGAUCCAC	2
>A06	GGAGCUCAGCCUUCACUGCUUGCCAUAGCGCGUAAGCUGCGUCGCCUGCUUCGGCAGU GCUGGUUUCGUCAGGUCGUGGCGAUGGCACCACGGUCGGAUCCAC	2
>A07	GGAGCUCAGCCUUCACUGCAGUGCAGGACUGCUAUUGAGGCCUGCUGCUUCGGAGCGU UCCGUGAUUCGAACUUGGGUUGAGGCACCACGGUCGGAUCCAC	2
>D12	GGAGCUCAGCCUUCACUGCCAAGGCGUAAGGAAGAGGCAAGAUACUCUUCGGCAGGACG UAACCGUGAUAUAUCAAGCAUCGGCACCACGGUCGGAUCCAC	2
>E06	GGAGCUCAGCCUUCACUGCUACGCUUCCAGAAGCAAUAUGCAGACUGCUUCGCAGUGAU AGAAGCGUUGCAACGCGGGAUGGGCACCACGGUCGGAUCCAC	2
>F07	GGAGCUCAGCCUUCACUGCAGGAAUAUUAAGCGUCGAAGGGUAUCCUGCUUCGGCAAAA UACGAAACCCCUCCUGUCAGUGACGGCACCACGGUCGGAUCCAC	2
>F08	GGAGCUCAGCCUUCACUGCUAAGCACCGAUUGAGACUGUGGAAUCUGCUUCGGCAGUG GGUGGUCAUCCAUUCCGUGUUGAGGCACCACGGUCGGAUCCAC	2
>A01	GGAGCUCAGCCUUCACUGCAACUGGUCCAAUGGCCGUCGCUGCCCUGCUUAGGCAGUC CGCGAGAUCGAGCGCGGCUCGAUAGGCACCACGGUCGGAUCCAC	1
>A02	GGAGCUCAGCCUUCACUGCACCAACCUGACUGGUGCAAGGGCUGCUGCUUCGGAGGGG AGAUCGUGAUGCUGUGACCGUGCGGCACCACGGUCGGAUCCAC	1
>A04	GGAGCUCAGCCUUCACUGCCAAAUGUCUCCGCGAGACAUUAUCUCCUGGCUCAGCAGGC GACAACGCGGACGCGGGCCGCACUGGCACCACGGUCGGAUCCAC	1
>A09	GGAGCUCAGCCUUCACUGCUGCAGUGCUCGAAAGCCGGCACUACUCUGCUUGGCAGCAC GAAGGACGUACGGUGCGGUAAUCGGCACCACGGUCGGAUCCAC	1
>A10	GGAGCUCAGCCUUCACUGCAGUGAAAAAAAUGUAAACAUAAGAUCUGCCUCGGCAGUCA CUGCAUCGACAACACCGUGGGAUGGCACCACGGUCGGAUCCAC	1
>A11	GGAGCUCAGCCUUCACUGCCCGGUAUCGUGUCACGCGGGGACACGCUGCUUCGGCAGU AUGACACAUCGACGCCCAUACUACCGGCACCACGGUCGGAUCCAC	1
>A12	GGAGCUCAGCCUUCACUGCAGUGAAAAAAAUGUAAACAUAAGAUCUGCUUCGGCAGUCA CUGCAUCGACAACACCGUGGGAUGGCACCACGGUCGGAUCCAC	1
>B01	GGAGCUCAGCCUUCACUGCAGUGAAAAAAAUGUAAACAUAAGAUCUGCUUCGGCAGUCA CUGCAUCGACAACACCGUGGAAUGGCACCACGGUCGGAUCCAC	1
>B03	GGAGCUCAGCCUUCACUGCCAAGCAGAUCGUUUUUGUCGGCCCGACCUGCUUCAGCAGU GCUCUACCGUGUGUGUCGUGCCUUCGGCACCACGGUCGGAUCCAC	1
>B05	GGAGCUCAGCCUUCACUGCUAUAGGCCAGUAUAGAAGUGAUGGCUCUGCUCCGGCCGUA GUGAGGACCCGAUUUGACCUGGUUGGCACCACGGUCGGAUCCAC	1
>B06	GGAGCUCAGCCUUCACUGCAGUGUGUGAAGACGGGCUCGACUAGGCUGAUUCGGCAGG UCCCCGUGUUGUUUUACGAGCUCUUGGCACCACGGUCGGAUCCAC	1
>B07	GGAGCUCAGCCUUCACUGCUGGAGCCGGAAUUUCUGAGCGAUUCCUGCUUCGGCAGAG GGGGUGAAUCCACUGUGGUAUGAAGGCACCACGGUCGGAUCCAC	1
>B09	GGAGCUCAGCCUUCACUGCCUUAUUCCGGACGCCGGGCCCGAAUGCUGCUACGGCAGU CGAAGACAACAUCGCGCCCUUCGGAGGCACCACGGUCGGAUCCAC	1
>B10	GGAGCUCAGCCUUCACUGCUGGAGCCGGAAUUUCUGAGCAAUUCCUGCUUCGGUAGAG GGGGUGAAUCCACUGUGGUGUGCAGGCACCACGGUCGGAUCCAC	1
>B12	GGAGCUCAGCCUUCACUGCAGCAAAGACUGGGAGCUCAUCUUCUGCUGCUUUGACAGGC GUGGAUUCUAAUCCCGACCGCUAUGGCACCACGGUCGGAUCCAC	1
>C01	GGAGCUCAGCCUUCACUGCUGACGAAAAAACCGUGAACGGAAUAUCUGCUUCGGCAGUG UCGGCGUGAUGAUGGGUUCAACCUGGCACCACGGUCGGAUCCAC	1
>C02	GGAGCUCAGCCUUCACUGCCAAAUGUCUCCGCGAGACAUUAUCACCUGGUUCGGCAGGC GACAACGCGGACGCGGGCCGCACUGGCACCACGGUCGGAUCCAC	1
>C04	GGAGCUCAGCCUUCCUGCUGCACCCAGGAAGGACUGUGGUGUCUCUGCUUUGGCAGUC CGUCGACGAAUACGCGGUUUUCGAGGCACCACGGUCGGAUCCAC	1
>C06	GGAGCUCAGCCUUCACUGCAGCAAAGACUGGGAGCUCAUCUUCUGCUGCUUCGACAGGC GUGGAUUCUAAUCCCGACCGCUAUGGCACCACGGUCGGAUCCAC	1
>C07	GGAGCUCAGCCUUCACUGCUAGCCAUAGCGCGUAAGCUGCGUCGCCUGCUUCGGCAGU GCUGGUUUCGUCAGGUCGUGGCGAUGGCACCACGGUCGGAUCCAC	1
>C08	GGAGCUCAGCCUUCACUGCCUUCGAGACGGUGUCACCGGAGCCGCUGCCUCGGCAGGA GGGAUUGUGGACCGCCCGUCUUGAGGCACCACGGUCGGAUCCAC	1
>C09	GGAGCUCAGCCUUCACUGCCUUAUUCCGGACGCCGGACCUGAGUGCUGCUUCGGCAGU CGAAGACAACAUCGCGCCCUUCGGAGGCACCACGGUCGGAUCCAC	1
>C10	GGAGCUCAGCCUUCACUGCGGUGAAGCUCCUUACGAUCAUUGCCUCUGCUUUGGCAGAC UCGCGGGAGGUUACGCGAUUCCUCGGCACCACGGUCGGAUCCAC	1

>C11	GGAGCUCAGCCUUCACUGCUACGCUUCCAGGAGCAGUAUGCAGACUGCUUCGCAGUGAU AGAAGCGUUGCAACGCAGGAUGGGCACCACGGUCGGAUCCAC	1
>D01	GGAGCUCAGCCUUCACUGCAGGAGUGGAGGUCAUUGGUCGACGACUGUUUCGGCAGGC AGCUGGGGCCUGUGGGUGCCGGUUGGCACCACGGUCGGAUCCAC	1
>D02	GGAGCUCAGCCUUCACUGCUCGGUAUCGUGUCACGCGGGGACGCGUUGCUUCGGCAGU AUGACACAUCGACGCCCAUACUACCGGACCACGGUCGGAUCCAC	1
>D03	GGAGCUCAGCCUUCACUGCAGGAGUGGUGGUUUUUGCUCCCCGUGCUGCUUCGGCCUC AAAUAAGUGACCGUUCGGGUCGAGGCACCACGGUCGGAUCCAC	1
>D04	GGAGCUCAGCCUUCACUGCAGGAAUAUUAAGCGUCGAAGGGUAUUCUGCUUCGGCAAAA UACGAAACCCCUCCUGUCAGUGACGGCACCACGGUCGGAUCCAC	1
>D05	GGAGCUCAGCCUUCACUGCCUUAUUCCGGACGCCGGACCCGAAUGCUGCUUCGGCAGU CGAAGACAACAUCGCGCCCUUCGGAGGCACCACGGUCGGAUCCAC	1
>D06	GGAGCUCAGCCUUCACUGCGCGAGUGAAAUUUAUAUGUCGUACGCGCUUCGGCAGCUGA CAACAGCCGUGGUGUGUGCCUUCGGCACCACGGUCGGAUCCAC	1
>D07	GGAGCUCAGCCUUCACUGCACCAACCUGACUGGUGCAAGGACUGCUGCUUCGGAGGGAA GAUCGUGAUGCUGUGACCGUGCGGCACCACGGUCGGAUCCAC	1
>D08	GGAGCUCAGCCUUCCUGCAUGGGCUGAAUCCUGGUCACACCGACUGUUCGGCAAGGCC GUGCCGUAAAGCCUUUCGUCCAAGGCACCACGGUCGGAUCCAC	1
>D09	GGAGCUCAGCCUUCACUGCUACGCUUCCAGAAGCAAUAAGCAGACUGCUUCGCAGUGAU AGAAGCGUUGCAACGCGGGAUGGGCACCACGGUCGGAUCCAC	1
>D10	GGAGCUCAGCCUUCACUGCUGGAGCCGGAAUUUCUGAGCAAAUCCUGCCUCGGCAGAG GGGGUGAAUCCACUGUGGUUUGAAGGCACCACGGUCGGAUCCAC	1
>D11	GGAGCUCAGCCUUCACUGCUGGGCGGAACCUCUUGAGCAAAGGUUCUGCUUCGGCAGU CGUGGUGUUAGGUCACUGAGCGCUUGGCACCACGGUCGGAUCCAC	1
>E01	GGAGCUCAGCCUUCACUGCUAGCAGGAUUCAGUGAUUGGUUUUGCAGCUUCGACAGUG CGCGUCCAAGUAAGGGCUCUCCUUGGCACCACGGUCGGAUCCAC	1
>E02	GGAGCUCAGCCUUCACUGCUGAGAGUGAUGGCUACUCCGCCAGGACUGCUUCGGCAGG AUGAGUCGUCCUGGUCUCGUGGUUAGGCACCACGGUCGGAUCCAC	1
>E03	GGAGCUCAGCCUUCACUGCAGGCACCACGGUCGGAUCCACAGUGGCGUAUGAAGGCACC ACGGUCGGAUCCAC	1
>E04	GGAGCUCAGCCUUCACUGCACUCCCGAAGAAAUCAGUGGUGUGUCUCUUCGGCAGCGU GUUGGCGGCCCCCGUGGUGUGCAGGCACCACGGUCGGAUCCAC	1
>E05	GGAGCUCAGCCUUCACUACUGGACUGCAGUAUCGCAGUGGCUGCUCUGCUGGCAGGUG GGGGUCGGAGCUGAUCGUGUUGAGGCACCACGGUCGGAUCCAC	1
>E07	GGAGCUCAGCCUUCACUGCAGGCACCACGGUCGGAUCCACAAUGUUCCGUGAAGGCACC ACGGUCGGAUCCACAGUUUGCAGUGUAGGCACCACGGUCGGAUCCAC	1
>E10	GGAGCUCAGCCUUCACUGCAAGCAGGAUACAGUGAUUGGUUUUGCGGCUUCGACAGUG CGCGUCCAAGUAAGGGCUCUCCUUGGCACACGGUCGGAUCCAC	1
>E12	GGAGCUCAGCCUUCACGCACUUGAACAGUCGAAGAAGUGAGCUCUCUUCGGCAGAACAA UGUUAGUGAAUUAACGUGGAUGGCACCACGGUCGGAUCCAC	1
>F01	GGAGCUCAGCCUUCACUGCUAGCAAGCUAACGCGUGAAGGUCAUUCUGCUACGGCAGCA AGCAUGCCGUUGCCAAGGAGUUCUGGCACCACGGUCGGAUCCAC	1
>F02	GGAGCUCAGCCUUCACUCAGGAAGCUCUAACGUCGUGAAGGGGACAGCUUCGACAGAGG CUACGUGGUGUCCGUGCAGGAUGGCACCACGGUCGGAUCCAC	1
>F03	GGAGCUCAGCCUUCACUGCUACGCUUCCAGAAGCAGUAUGCAGACUGCUUCGCAGUGAU AGAAGCGUUGCAACGCGGGAUGGGCACCACGGUCGGAUCCAC	1
>F04	GGAGCUCAGCCUUCACUGCUACGCUUCCAGAAGCAAUAUGCAGAUUGCUUCGCAGUGAU AGAAGCGUUGCAACGCGGGAUAGGCACCACGGUCGGAUCCAC	1
>F05	GGAGCUCAGCCUUCAUGCGGACCACUGAAACAGUCAGGUUGCCUCUGUUUCGGCAGGC GUUUUGACUCGGCCGCCUCCACGUGGCACCACGGUCGGAUCCAC	1
>F06	GGAGCUCAGCCUUCACUGCCUUGUUGUGGCGAGGUACACAAUAAACUGCUCCGGCAGGC GGGGGCUAACCGACCGGUAUACCAGGCACCACGGUCGGAUCCAC	1
>F09	GGAGCUCAGCCUUCACUGCCUUAUUCCGGACGCCGGGCCCGAGUGCUGCUUCGGCAGU CGAAGAUAACAUCGCGCCCUUCGGAGGCACCACGGUCGGAUCCAC	1
>F10	GGAGCUCAGCCUUCACUGCUAUAGGCCAGUAUAGAAGCGAUGGCUCUGCUUCGGCCGUA GUGAGGACCCGAUUUGACCUGUUUGGCACCACGGUCGGAUCCAC	1
>F12	GGAGCUCAGCCUUCACUGCUGGGCAGAACCUCUUGAGCAAAGGUUCUGCUUCGGCAGU CGUGGUGUUAGGUCACUGAGCGCUUGGCACCACGGUCGGAUCCAC	1
>G02	GGAGCUCAGCCUUCACUGCUGACGAAAAAACCGUGAACGGAAUAUCUGCCUCGGCAGUG UCGGCGUGAUGAUGGGUUCAACCUGGCACCACGGUCGGAUCCAC	1
>G03	GGAGCUCAGCCUUCACUGCAAGCAGGAUUCAGUGAUUGGUUUUGCGGCUUCGGCAGUG CGCGUCCAAGUAAGGGCUCUCCUUGGCACCACGGUCGGAUCCAC	1
>G04	GGAGCUCAGCCUUCACUGCAUGCACCACGGUCGGAUCCACAGUGGCUUGAAGGCACCAC GGUCGGAUCCACACGUGUUCUUGAAGGCACCACGGUCGGAUCCAC	1
>G06	GGAGCUCAGCCUUCACUCAACGGGUGGUAUGUGGAGUGCUGUCUCCGCUUCGGCAGAU UGAAGGAAGGGAUGCGACCGCUAUGGCACCACGGUCGGAUCCAC	1

>G07	GGAGCUCAGCCUUCCUGCUCUCCGUGCAAUCCGACCUUAACUUCUGCUUCGGCAGAGAG GAGCCACUGGUAGCUCCGCUUUGGCACCACGGUCGGAUCCAC	1
>G09	GGAGCUCAGCCUUCACUGCUGGGCGGAACCCCUUGAGCAAAGGUUCUGCUUCGGCAGU CGUGGUGUUAGGUCACUGAGCGCUUGGCACCACGGUCGGAUCCAC	1
>H03	GGAGCUCAGCCUUCCUGCUAAGUGCGGGUGCUUUACGUGGUGUGCUGCUUCGGCAGCC UCGUCGGGCCUUACGCGAUCUGGUGGCACCACGGUCGGAUCCAC	1
>H04	GGAGCCGGAAUUUCUGAGCAAUUCCUGCUUCGGCAGAGGGGGUGAAUCCACUGUGGUG UGCAGGCACCACGGUCGGAUCCACAUGGUUCGAAGGCACCACGGUCGGAUCCAC	1
>H05	GGAGCUCAGCCUUCACUGCAGCUGGCAAAAUGCAGCUCACCUCUGUYUCGGCAGGGGAA AUACAUUAAGCGAUUCGUGUUGGCACCACGGUCGGAUCCAC	1
>H06	GGAGCUCAGCCUUCACUGCUGGCGCCGGAAUUUCUGAGCAAUUCCCGCUUCGGCAGAG UGGGUGAAUCCACUGUGGUCUGAAGGCACCACGGUCGGAUCCAC	1
>H07	GGAGCUCAGCCUUCACUGCGGUGUCUGCGGUUCUGGCGUGCAUUCCUGCUUGCAGGAC AGGAGUUGCGACCAGUAUCGUUUGGCACCACGGUCGGAUCCAC	1
>H08	GGAGCUCAGCCUUCACUGCACCAACCUGCCUGGUGCAAGGACUGUUGCUUCGGAGGGA AGAUCGUGAUGCUGUGACCGUGCGGCACCACGGUCGGAUCCAC	1
>H09	GGAGCUCAGCCUUCACUGCUGGAGCCGGAAUUUCUGAGCAAUUCCUGCUUCGGCAGAGA GGGUGAAUCCACUGUGGUGUGCAGGCACCACGGUCGGAUCCAC	1
>H10	GGAGCUCAGCCUUCACUGCAGGAAUAUUAAGCGUCGAAGGGUAUCCUGCUUCGGCAAAA UACGAAACCCCUCCUGCCAGUGACGGCACCACGGUCGGAUCCAC	1
>H11	GGAGCUCAGCCUUCACUGCAGCUGGCAAAAUGCAGCUCACCCCUGUUUUCGGCAGGGGA AAUACAUUAAGCGAUUCGUGUUGGCACCACGGUCGGAUCCAC	1
>H12	GGAGCUCAGCCUUCACUCUGUUGCCGACAGGACAGGCUGGACUCUGCUUCGAAGGCCG CUUCCGUGCCAGCGCCAGGAUGGCCCACGGUCGGAUCCAC	1

Supplementary Table S3: Sequences used for *SELEX*, *in vitro* and *in vivo* transcription of the aptamers

Name	Sequences
**Forward primer**	TCTAATACGACTCACTATAGGAGCTCAGCCTTCACTGC
**Reverse primer (Primer B)**	GTGGATCCGACCGTGGTGCC
**DNA library sequence**	GTGGATCCGA CCGTGGTGCC NNNNNNNNNNNNNNNNNNNNNNNNNN CTG CCG AAG CAG NNNNNNNNNNNNNNNNNNNNNNNNNN GCAGTGAAGG CTGAGCTCC
**RNA library sequence**	GGAGCUCAGC CUUCACUGC NNNNNNNNNNNNNNNNNNNNNNNNNN CUGCUUCGGCAG NNNNNNNNNNNNNNNNNNNNNNNNNN GGCACCACGG UCGGAUCCAC
**Clone 5**	TAATACGACTCACTATA GGAGCTCAGC CTTCACTGCG GTTGCCCTCC GTGCTTCATG CTTCCCTGCT TCGGCAGTCG CGGTAAACCG TAGAGAAAGT TGAGGCACCA CGGTCGGATC CAC
**DNB**	TAATACGACTCACTATA GGTGCCTTAT TCCGGACGCC GGGCCCGAAT GCTGCTACGG CAGTCGAAGA CAACATCGCG CCCTTCGGAG GCACC
**wt-DNB**	TAATACGACTCACTATA GGAGCTCAGC CTTCACTGCC TTATTCCGGA CGCCGGGCCC GAATGCTGCT ACGGCAGTCG AAGACAACAT CGCGCCCTTC GGAGGCACCA CGGTCGGATC CAC
**pET28-tRNA**	…CGATCCCGCGAAAT TAATACGACTCACTATA GGG GCCCGGATAG CTCAGTCGGT AGAGCAG CGGCCG CGGGTCCAGG GTTCAAGTCC CTGTTCGGGC GCCA TAGCATAACC CCTTGGGGCC TCTAAACGGG TCTTGAGGGG TTTTTTG CTCGAG…
**pET28-SRB-2**	…CGATCCCGCGAAAT TAATACGACTCACTATA GGG GCCCGGATAG CTCAGTCGGT AGAGCAG CGGCCG ACCTCGC TTCGGCGATG ATGGAGAGGC GCAAGGTTAA CCGCCTCAGG T CGGCCG CGGGTCCAGG GTTCAAGTCC CTGTTCGGGC GCCA TAGCATAACC CCTTGGGGCC TCTAAACGGG TCTTGAGGGG TTTTTTG CTCGAG…
**pET28-DNB**	…CGATCCCGCGAAAT TAATACGACTCACTATA GGG GCCCGGATAG CTCAGTCGGT AGAGCAG CGGCCG GGTGCCTTAT TCCGGACGCC GGGCCCGAAT GCTGCTACGG CAGTCGAAGA CAACATCGCG CCCTTCGGAG GCACC CGGCCG CGGGTCCAGG GTTCAAGTCC CTGTTCGGGC GCCA TAGCATAACC CCTTGGGGCC TCTAAACGGG TCTTGAGGGG TTTTTTG CTCGAG…
**pET28-SRB-2-DNB**	…CGATCCCGCGAAAT TAATACGACTCACTATA GGG GCCCGGATAG CTCAGTCGGT AGAGCAG CGGCCG ACCTCGCTTC GGCGATGAT GGAGAGGCGC AAGGTTAACC GCCTCAGGT CGGCCG CGGGTCCAGGGTT CAAGTCCCTG TTCGGGCGCCA TAGCATAAC CCCTTGGGGC CTCTAAACGG GTCTTGAGGG GTTTTTTG CTCGAG CACCAC CACCACCACC ACTGAGATCC GGCTGCTAAC AAAGCCCGAA AGGAAGCTGA GTTGGC TAATACGACTCACTATA GGG GCCCGGATAG CTCAGTCGGT AGAGCAG CGGCCG GGTGCCTTAT TCCGGACGCC GGGCCCGAAT GCTGCTACGG CAGTCGAAGA CAACATCGCGC CCTTCGGAG GCACC CGGCCG CGGGTCCAGG GTTCAAGTCC CTGTTCGGGCGCCA TAGCAT AACCCCTTGG GGCCTCTAAA CGGGTCTTGA GGGGTTTTTTG CTCGAG CACCACCTGA AAGGAGGAAC…

**Legend**	
**Purple**	T7 Promoter
**Orange**	T7 Terminator
**Red**	SRB-2 aptamer
**Green**	DNB aptamer
**Blue**	tRNA scaffold

E) Supplementary References

1. Cadwell, R.C. and Joyce, G.F. (1994) Mutagenic PCR. *PCR Methods Appl.*, **3**, S136-140.
2. Zuker, M. (2003) Mfold web server for nucleic acid folding and hybridization prediction. *Nucleic Acids Res.*, **31**, 3406-3415.
3. Davis, J.H. and Szostak, J.W. (2002) Isolation of high-affinity GTP aptamers from partially structured RNA libraries. *Proc. Natl. Acad. Sci. U. S. A.*, **99**, 11616-11621.
